# Supplementary material for: Silver Catalyzed Site-Selective C(sp3)−H Bond Amination of Secondary over Primary C(sp3)−H Bonds
Source: Molecules. 2022 Sep 21;27(19):6174. doi: 10.3390/molecules27196174 (PMC9571442; doi:10.3390/molecules27196174)

Supporting Information

Silver catalyzed Site-selective C(sp^3)−H Bond Amination of Secondary over Primary C(sp^3)−H Bonds

Luzhen Jiao, Dawei Teng, Zixuan Wang and Guorui Cao *

State Key Laboratory Base of Eco-Chemical Engineering, College of Chemical Engineering, Qingdao University of Science and Technology, Qingdao 266042, China.

Table of Contents

1. Preparation and characterization of substrates **1-13**……………………………………S2-S3

2. ^{1}H NMR and ^{13}C NMR spectra of new compounds……………………….…………S3-S40

*Corresponding author. e-mail: caoguorui@qust.edu.cn

1. Preparation and characterization of substrates 1-13

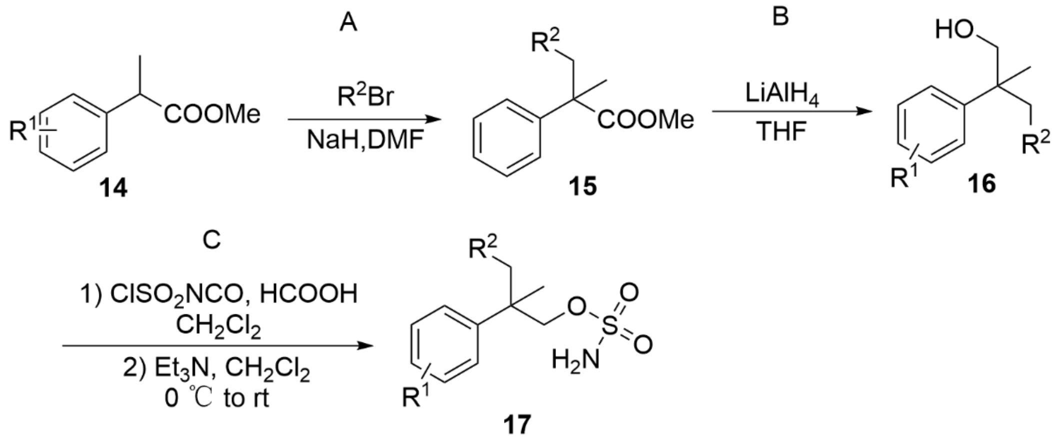

General procedure: A) Under argon atmosphere, NaH (13.4 mmol) was added to a solution of carboxylic acid ester (12.18 mmol) in THF (50 ml) at 0°C. The stirring was applied at 0°C until complete formation of anion. R^2Br was slowly added at 0°C and under argon atmosphere, then the reaction mixture was warmed to room temperature, and stirred until complete disappearance of starting material (10 hours). The reaction was quenched with NH_4Cl (0.2 ml), and extracted with EtOAc. The organic layer was washed with aqueous saturated thiosulfate solution, water then dried on sodium sulphate. The residue was subjected to column chromatography on silica gel (200-300 mesh) using petroleum/ethyl acetate as eluent to afford **15**.

B) To a solution of **15** (10 mmol) in THF (20 ml) was added $LiAlH_4$ (1.14 g, 30 mmol) in THF (40ml) slowly at 0 °C. Then stir the reaction mixture for addition 30 minutes. The reaction mixture was warmed slowly to room temperature (25 °C) and stirred overnight. Until the **15** was completely consumed (monitored by TLC), the reaction was quenched by adding H_2O (15 mL). The organic phase was collected and the aqueous layer was extracted with EtOAc (3 × 15 mL). Then combined organic layers were washed with brine, dried over Na_2SO_4, and concentrated under reduced pressure. The crude mixture was purified by flash column chromatography on silica gel (200 ~ 300 mesh) to give the desired sulfamate ester **16**.

C) To a 50 mL round-bottom flask equipped with a stirring bar and rubber septum was added a solution of $ClSO_2NCO$ (2.0 equiv, 2 mol/L) in CH_2Cl_2 under argon. Then neat

formic acid (2.0 equiv) was added dropwise at 0 °C. After the **16** addition, the reaction mixture was warmed slowly to room temperature (25 °C) and stirred overnight. After cooling the mixture back to 0 °C, a solution of alcohol (1.0 equiv, 1 mol/L) and Et_3N (2.0 equiv) in CH_2Cl_2 were added dropwise. Then the mixture was stirred for 30 min at 0 °C. When the alcohol was completely consumed (monitored by TLC), the reaction was quenched by adding H_2O (15 mL) until a clear solution was formed. The organic phase was collected and the aqueous layer was extracted with CH_2Cl_2 (3 × 15 mL). The combined organic layers were washed with brine, dried over Na_2SO_4, and concentrated under reduced pressure. The crude mixture was purified by flash column chromatography on silica gel (200 ~ 300 mesh) to give the desired sulfamate ester **17**.

2. ^{1}H NMR and ^{13}C NMR spectra of new compounds

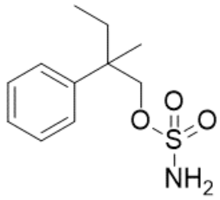

2-methyl-2-phenylbutyl sulfamate (**1**)

^{1}H NMR (500 MHz, DMSO-d6) δ 7.37 (s, 2H), 7.29 – 7.22 (m, 4H), 7.14 (tt, J = 5.6, 2.6 Hz, 1H), 4.04 (d, J = 9.4 Hz, 1H), 3.98 (d, J = 9.5 Hz, 1H), 1.67 (dt, J = 14.7, 7.3 Hz, 1H), 1.54 (dq, J = 14.5, 7.4 Hz, 1H), 1.22 (s, 3H), 0.55 (t, J = 7.4 Hz, 3H). ^{13}C NMR (126 MHz, DMSO-d6) δ 142.25, 126.62, 124.77, 124.53, 74.19, 39.73, 29.11, 20.04, 6.47.; HRMS (ESI-TOF$^+$): m/z Calcd. for $C_{11}H_{18}NO_3S$ [$(M+H)^+$]: 244.1007. Found: 244.1011

^{1}H NMR Spectrum of 1

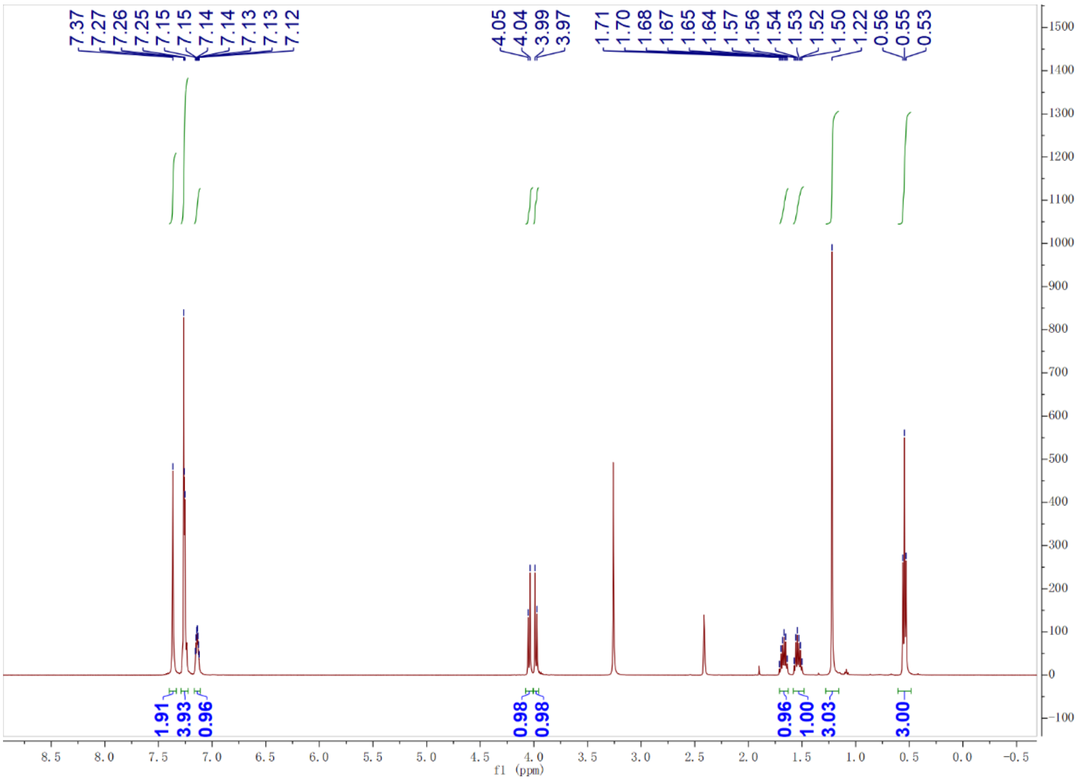

^{13}C NMR Spectrum of 1

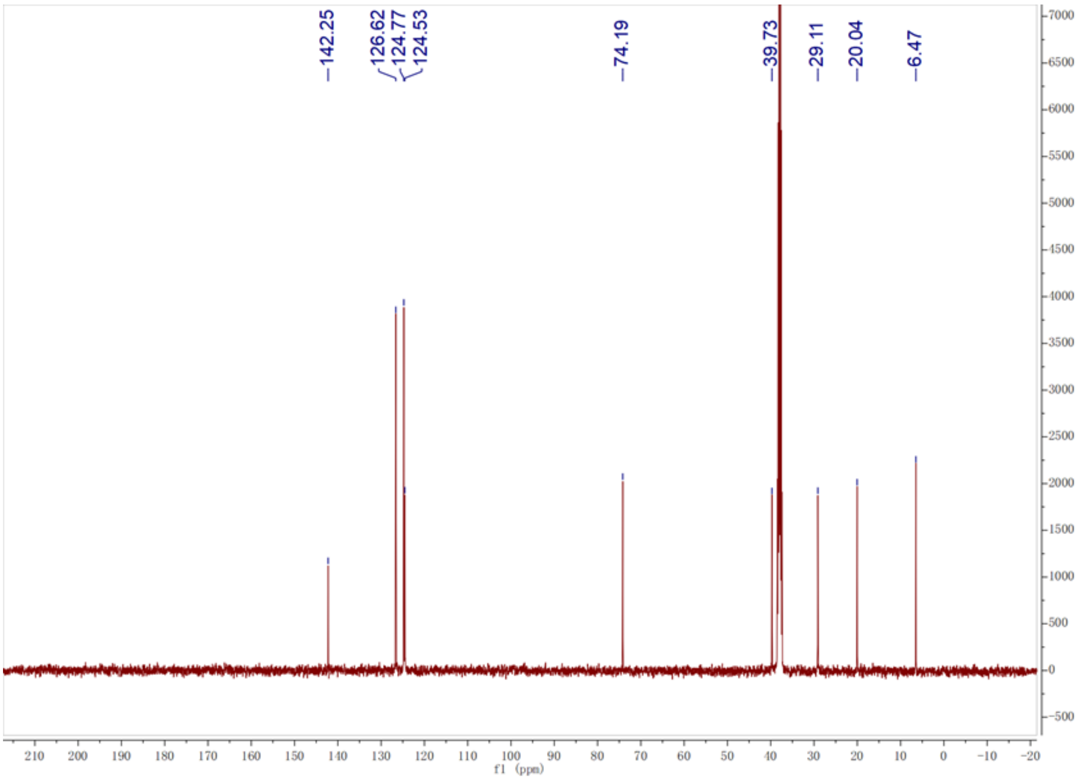

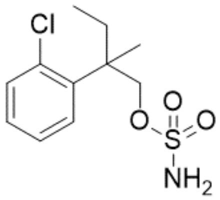

2-(2-chlorophenyl)-2-methylbutyl sulfamate（**2**）

^{1}H NMR (500 MHz, DMSO-d6) δ 7.40 (s, 2H), 7.31 (ddd, J = 13.1, 7.8, 1.6 Hz, 2H), 7.21 (dtd, J = 22.6, 7.4, 1.6 Hz, 2H), 4.50 (d, J = 9.4 Hz, 1H), 4.11 (d, J = 9.5 Hz, 1H), 2.13 (dd, J = 14.2, 7.3 Hz, 1H), 1.64 (dd, J = 14.1, 7.3 Hz, 1H), 1.37 (s, 3H), 0.53 (t, J = 7.5 Hz, 3H). ^{13}C NMR (126 MHz, DMSO-d6) δ 139.27, 132.28, 131.68, 130.46, 128.42, 127.18, 73.68, 43.12, 22.65, 8.28.; HRMS (ESI-TOF$^+$): m/z Calcd. for $C_{11}H_{17}ClNO_3S$ [(M+H)$^+$]: 278.0618. Found: 278.0615

^{1}H NMR Spectrum of 2

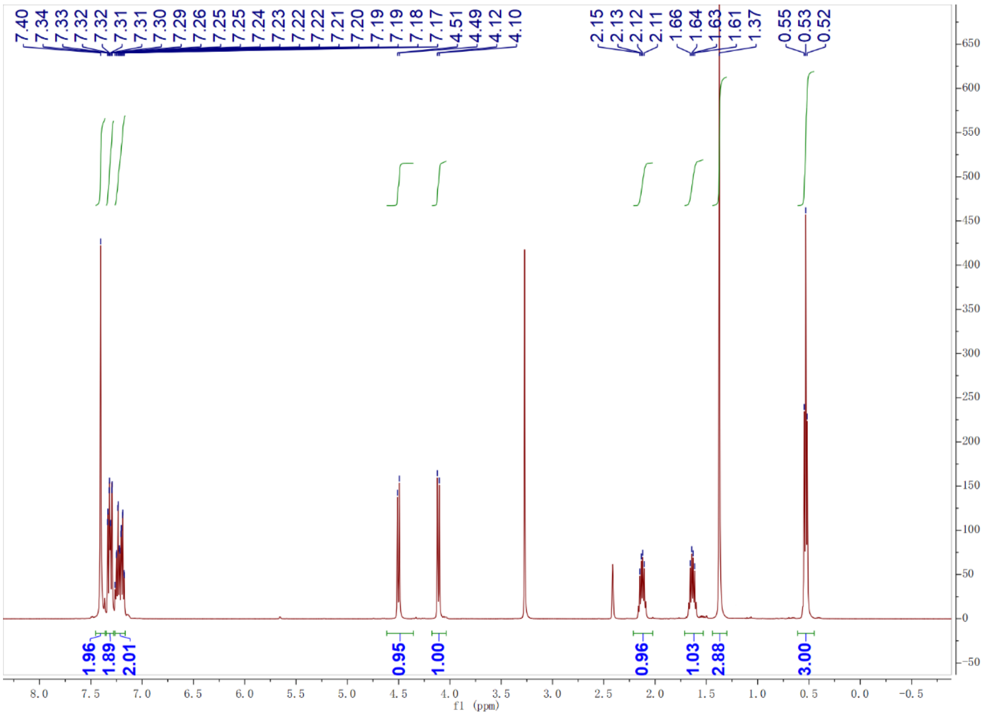

^{13}C NMR Spectrum of 2

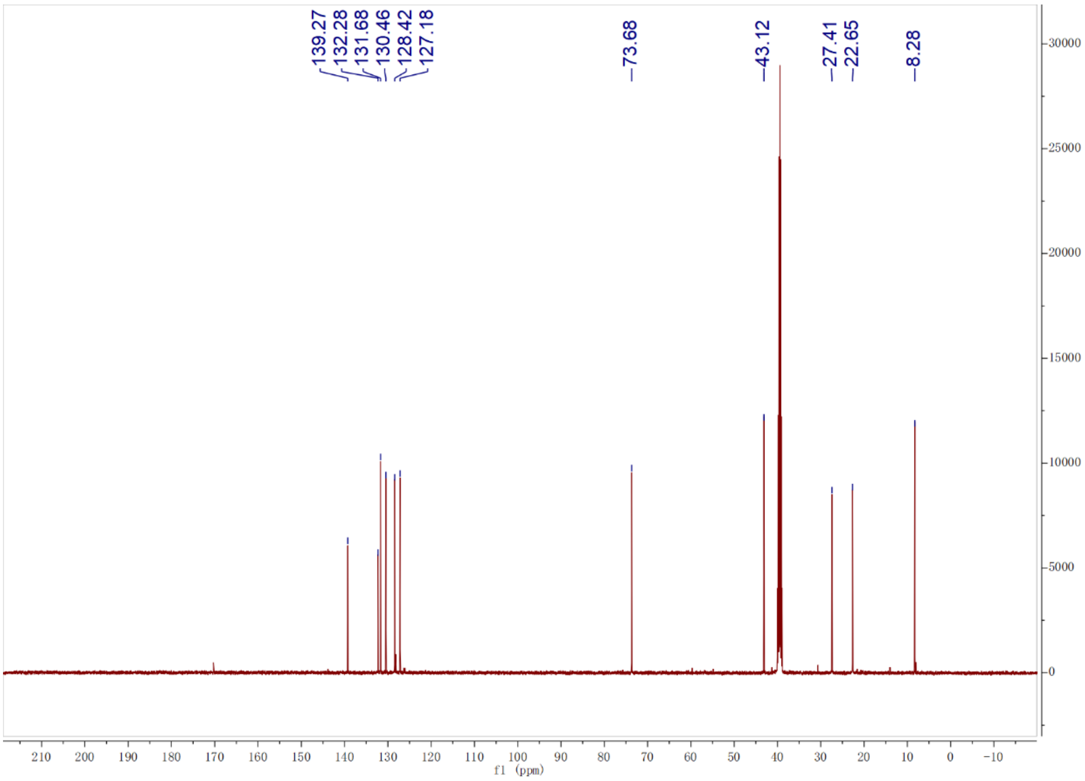

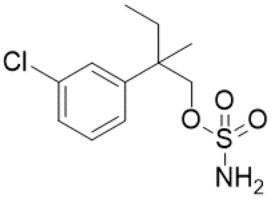

2-(3-chlorophenyl)-2-methylbutyl sulfamate（**3**）

^{1}H NMR (500 MHz, DMSO-d6) δ 7.38 (d, J = 2.3 Hz, 2H), 7.29 (dd, J = 5.0, 3.4 Hz, 2H), 7.24 (dt, J = 8.0, 1.5 Hz, 1H), 7.22 – 7.20 (m, 1H), 4.06 (d, J = 9.5 Hz, 1H), 3.97 (d, J = 9.9 Hz, 1H), 1.67 (dq, J = 14.7, 7.4 Hz, 1H), 1.52 (dd, J = 14.1, 7.3 Hz, 1H), 1.22 (s, 3H), 0.55 (t, J = 7.4 Hz, 3H). ^{13}C NMR (126MHz, DMSO-d6) δ 146.65, 133.09, 129.99, 126.44, 126.20, 125.24, 75.44, 41.58, 30.53, 7.95.; HRMS (ESI-TOF^{+}): m/z Calcd. for $C_{11}H_{17}ClNO_3S$ [(M+H)$^{+}$]: 278.0618. Found: 278.0619

^{1}H NMR Spectrum of 3

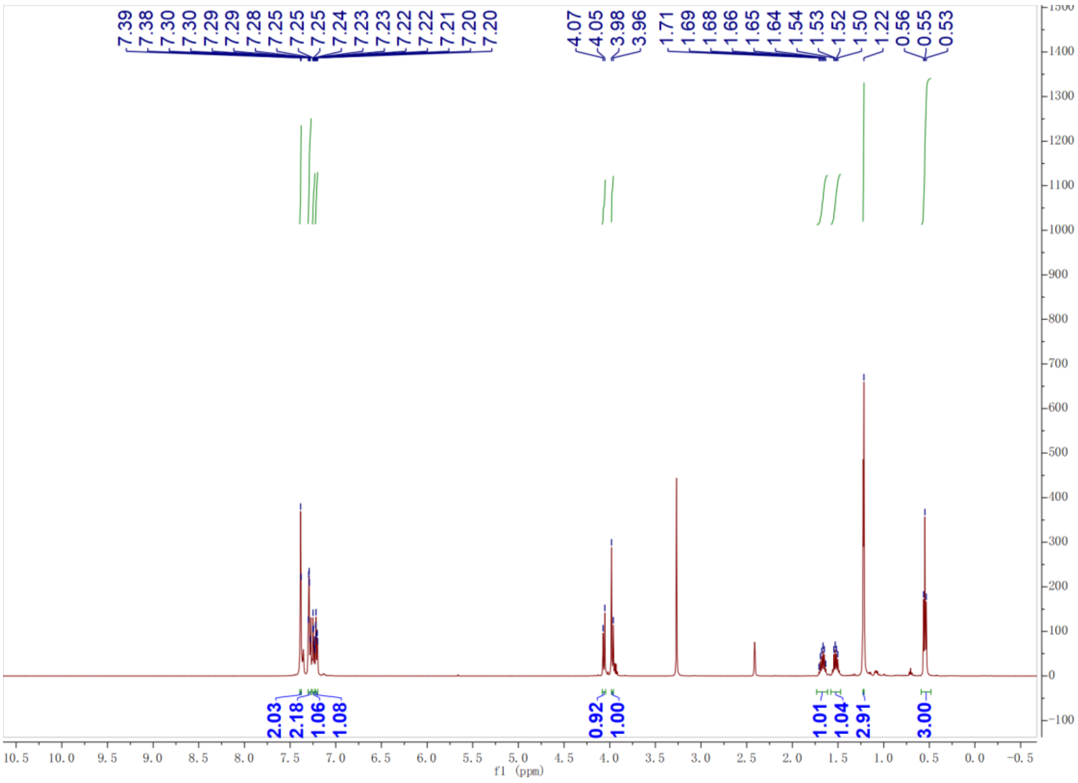

^{13}C NMR Spectrum of 3

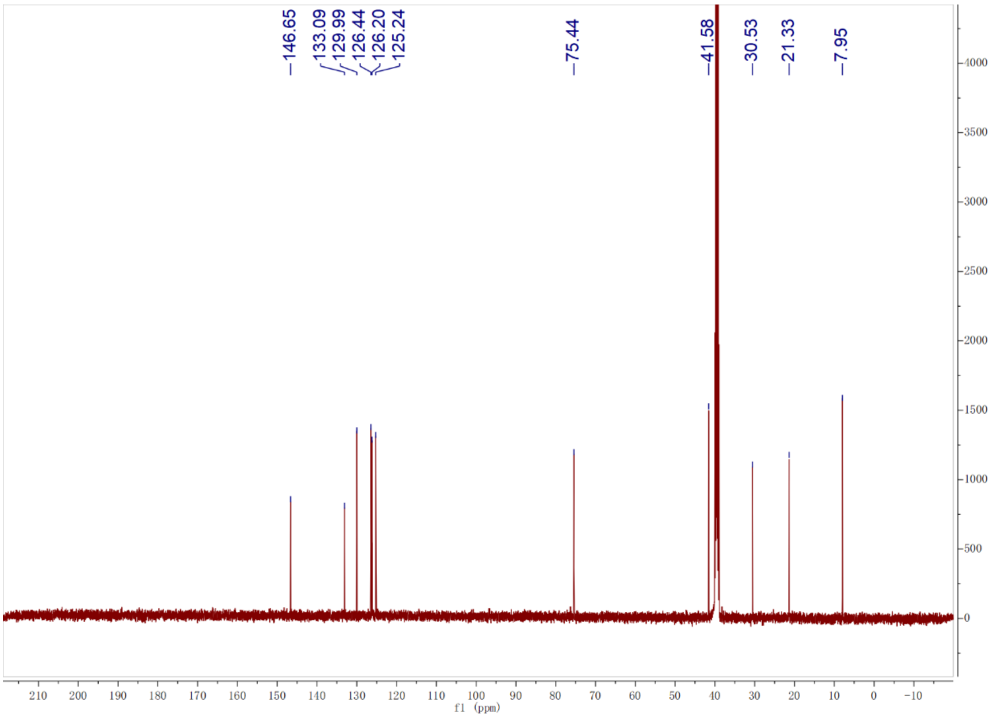

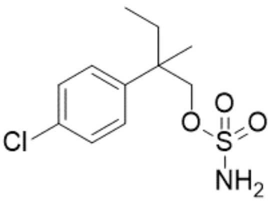

2-(4-chlorophenyl)-2-methylbutyl sulfamate（**4**）

^{1}H NMR (500 MHz, DMSO-d6) δ 7.38 (s, 2H), 7.29 (d, J = 2.4 Hz, 4H), 4.05 (d, J = 9.7 Hz, 1H), 3.96 (d, J = 9.3 Hz, 1H), 1.65 (dt, J = 14.9, 7.4 Hz, 1H), 1.52 (dq, J = 14.6, 7.4 Hz, 1H), 1.21 (s, 3H), 0.54 (t, J = 7.4 Hz, 3H). ^{13}C NMR (126 MHz, DMSO-d6) δ 142.90, 130.83, 128.43, 128.05, 75.55, 41.21, 30.57, 21.40, 7.93.; HRMS (ESI-TOF^{+}): m/z Calcd. for $C_{11}H_{17}ClNO_3S$ $[(M+H)^{+}]$: 278.0618. Found: 278.0618

^{1}H NMR Spectrum of 4

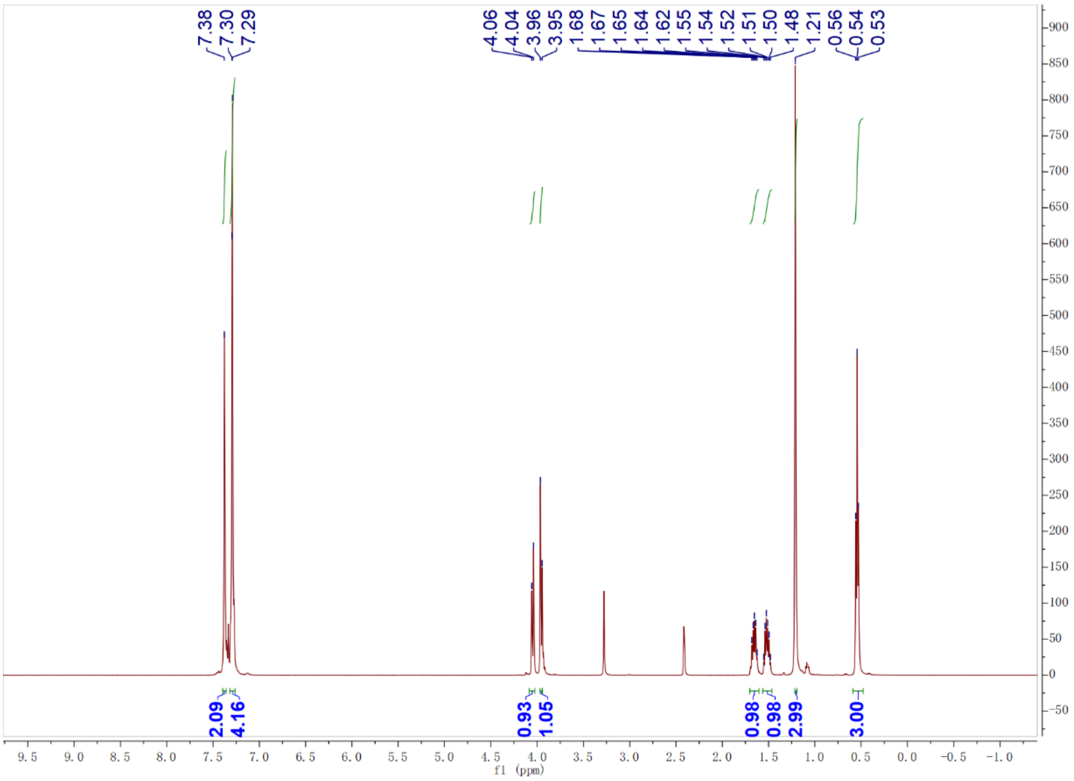

^{13}C NMR Spectrum of 4

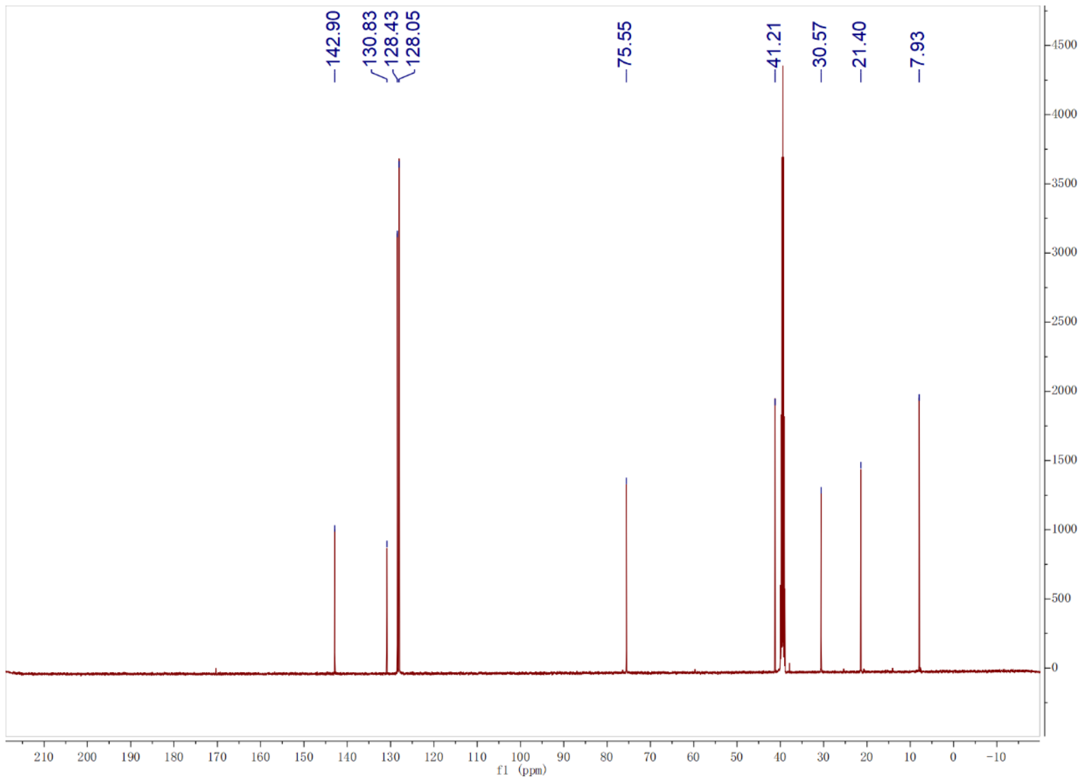

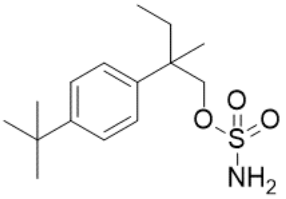

2-(4-(tert-butyl)phenyl)-2-methylbutyl sulfamate（**5**）

^{1}H NMR (500 MHz, DMSO-d6) δ 7.36 (s, 2H), 7.28 – 7.24 (m, 3H), 7.19 (s, 1H), 4.01 (d, J = 9.4 Hz, 1H), 3.96 (d, J = 9.4 Hz, 1H), 1.65 (dd, J = 14.0, 7.3 Hz, 1H), 1.53 (dt, J = 13.8, 7.2 Hz, 1H), 1.20 (s, 3H), 1.19 (s, 9H), 0.56 (t, J = 7.4 Hz, 3H). ^{13}C NMR (126 MHz, DMSO-d6) δ 148.18, 140.76, 125.97, 124.91, 75.72, 40.84, 33.97, 31.10, 30.59, 21.68, 8.12.; HRMS (ESI-TOF$^+$): m/z Calcd. for $C_{15}H_{26}NO_3S$ [(M+H)$^+$]: 300.1633. Found: 300.1634

^{1}H NMR Spectrum of 5

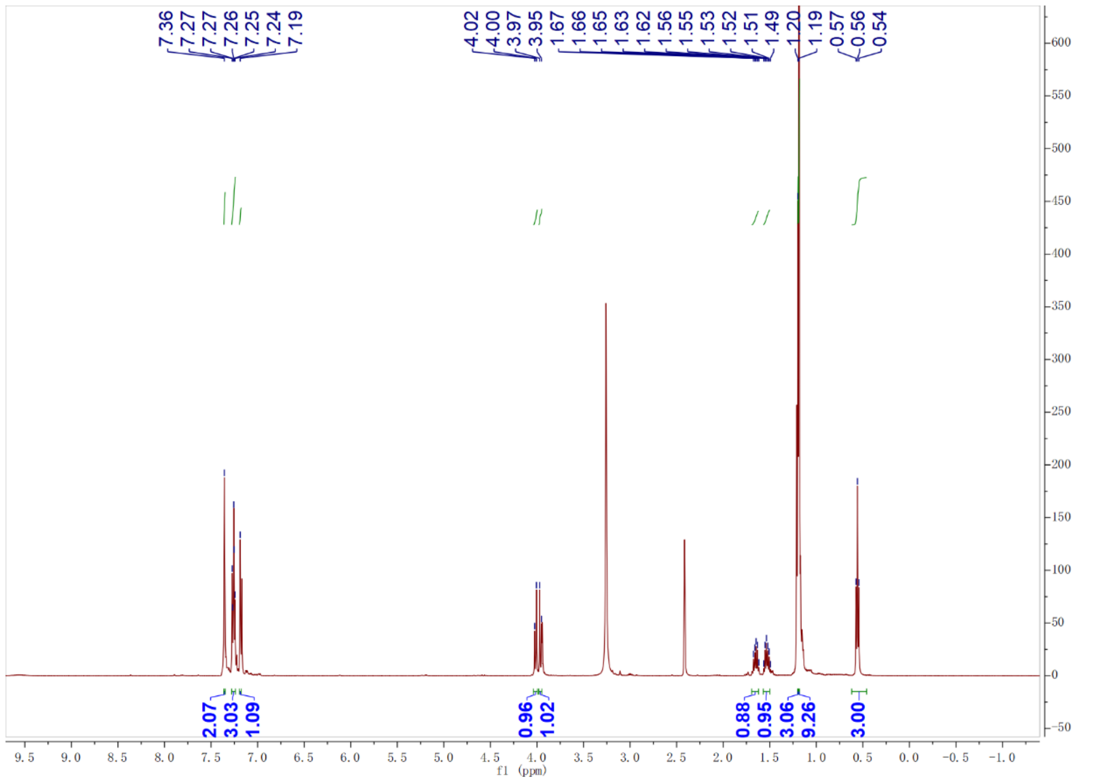

^{13}C NMR Spectrum of 5

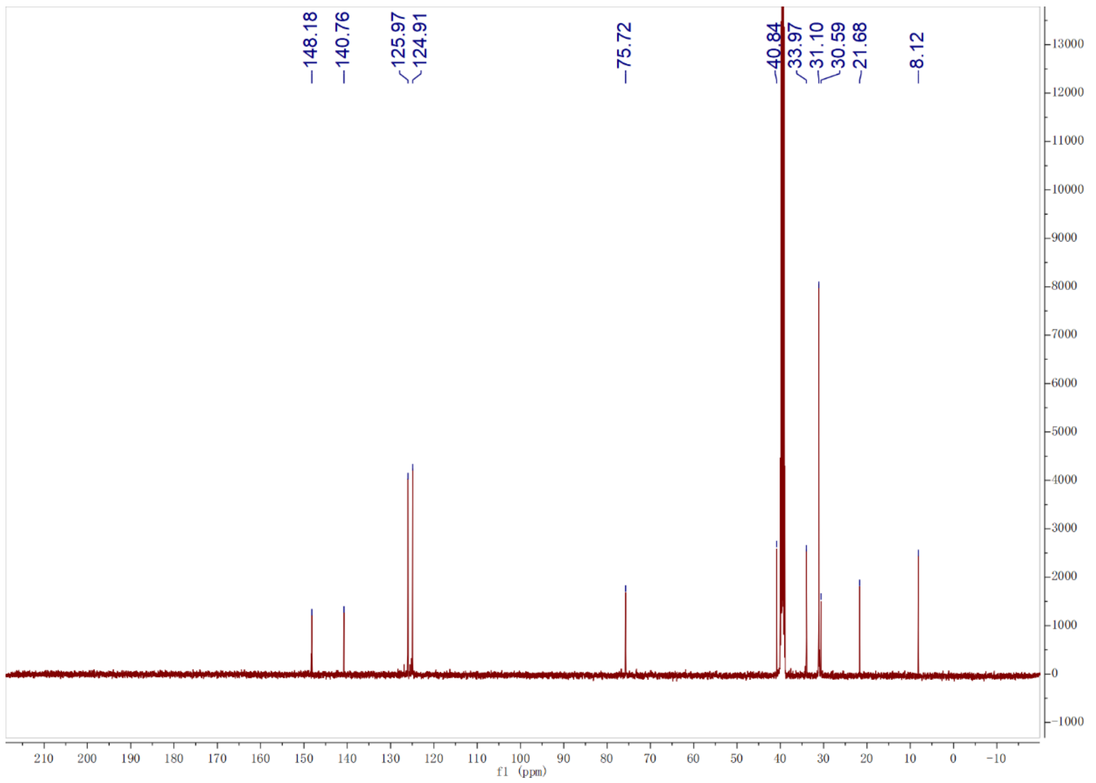

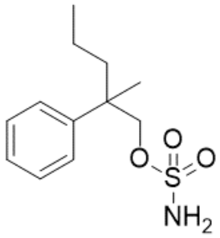

2-methyl-2-phenylpentyl sulfamate（**6**）

^{1}H NMR (500 MHz, DMSO-d6) δ 7.36 (s, 2H), 7.26 (d, J = 6.5 Hz, 4H), 7.13 (tt, J = 6.4, 2.0 Hz, 1H), 4.03 (d, J = 9.4 Hz, 1H), 3.97 (d, J = 9.3 Hz, 1H), 1.60 (td, J = 12.9, 4.4 Hz, 1H), 1.48 (td, J = 13.4, 12.9, 4.6 Hz, 1H), 1.24 (s, 3H), 1.05 – 0.96 (m, 1H), 0.86 (ddd, J = 19.6, 9.8, 6.0 Hz, 1H), 0.71 (t, J = 7.2 Hz, 3H). ^{13}C NMR (126 MHz, DMSO-d6) δ 144.12, 128.18, 126.20, 126.07, 75.98, 41.13, 40.61, 22.11, 16.60, 14.46.; HRMS (ESI-TOF$^+$): m/z Calcd. for $C_{12}H_{20}NO_3S$ [(M+H)$^+$]: 258.1164. Found: 258.1165

^{1}H NMR Spectrum of 6

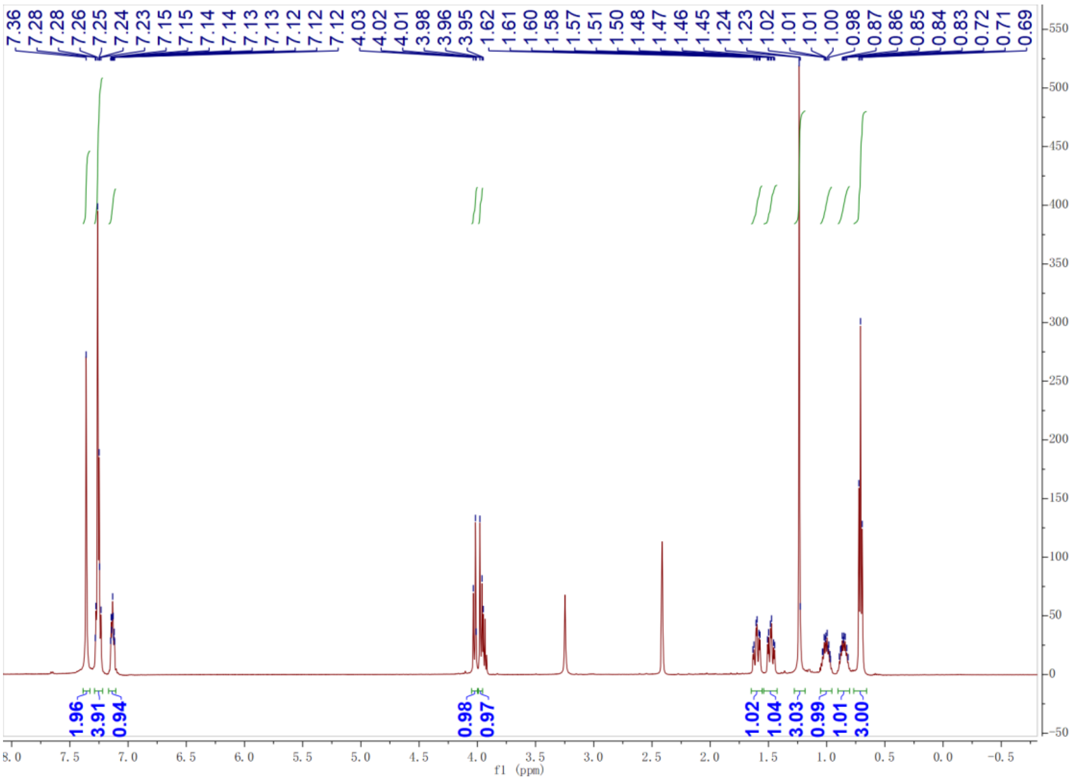

^{13}C NMR Spectrum of 6

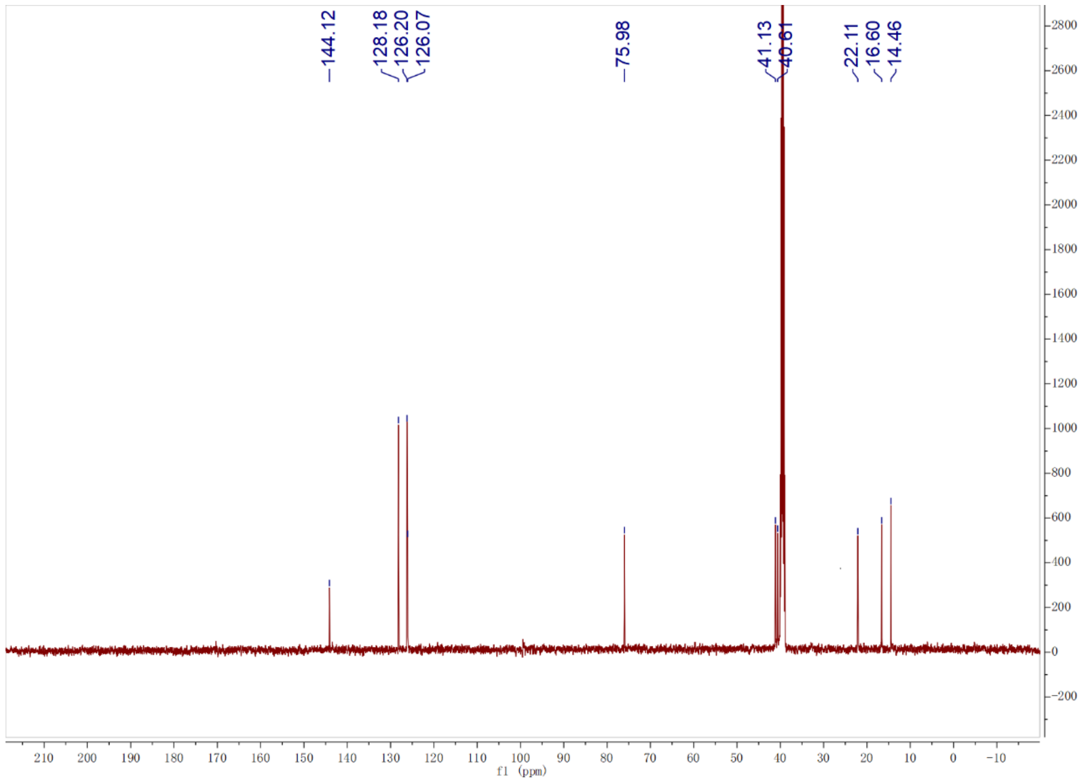

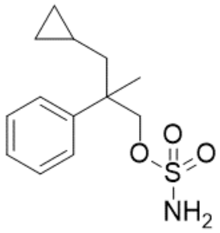

3-cyclopropyl-2-methyl-2-phenylpropyl sulfamate（**7**）

^{1}H NMR (500 MHz, DMSO-d_6) δ 7.37 (s, 2H), 7.33 – 7.29 (m, 2H), 7.25 (t, *J* = 7.8 Hz, 2H), 7.14 (d, *J* = 7.1 Hz, 1H), 4.15 (d, *J* = 9.4 Hz, 1H), 4.06 (d, *J* = 9.4 Hz, 1H), 1.62 (dd, *J* = 14.0, 5.9 Hz, 1H), 1.39 (dd, *J* = 14.0, 6.9 Hz, 1H), 1.32 (s, 3H), 0.34 – 0.15 (m, 3H), -0.04 – -0.13 (m, 1H), -0.13 – -0.21 (m, 1H). ^{13}C NMR (126 MHz, DMSO-d6) δ 144.61, 128.11, 126.24, 126.05, 75.44, 43.51, 42.11, 22.50, 6.04, 4.79, 4.06.; HRMS (ESI-TOF$^+$): m/z Calcd. for $C_{13}H_{22}NO_3S$ [(M+H)$^+$]: 272.1320. Found: 272.1322

^{1}H NMR Spectrum of 7

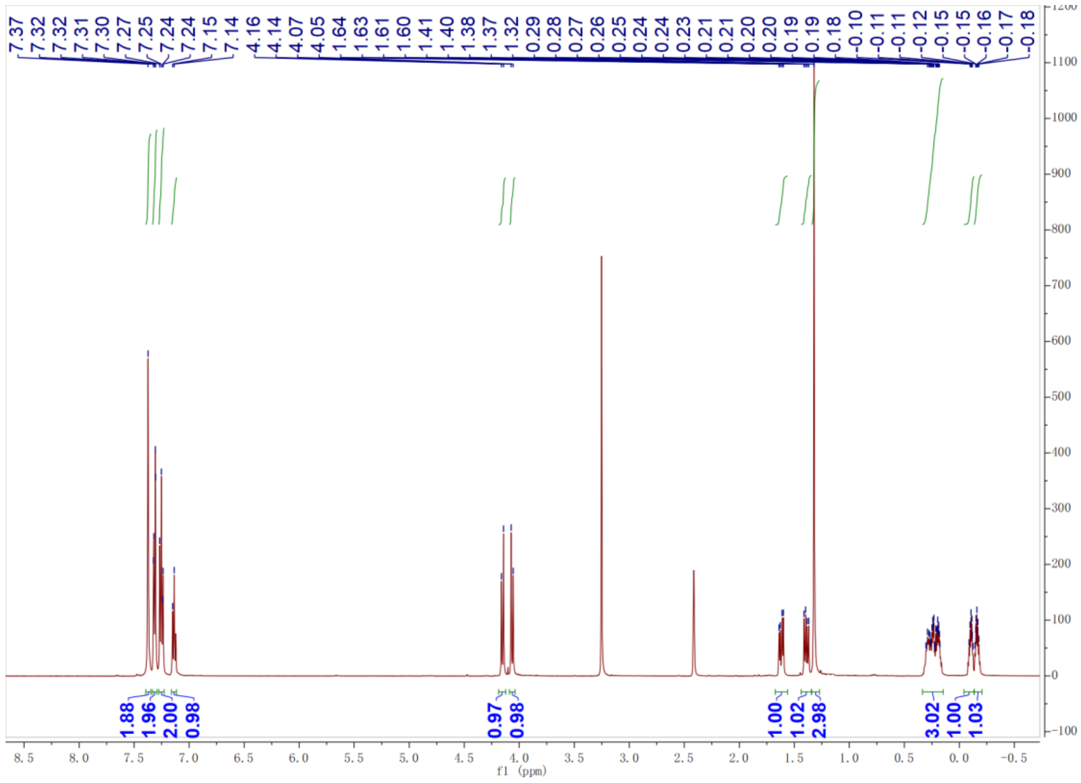

^{13}C NMR Spectrum of 7

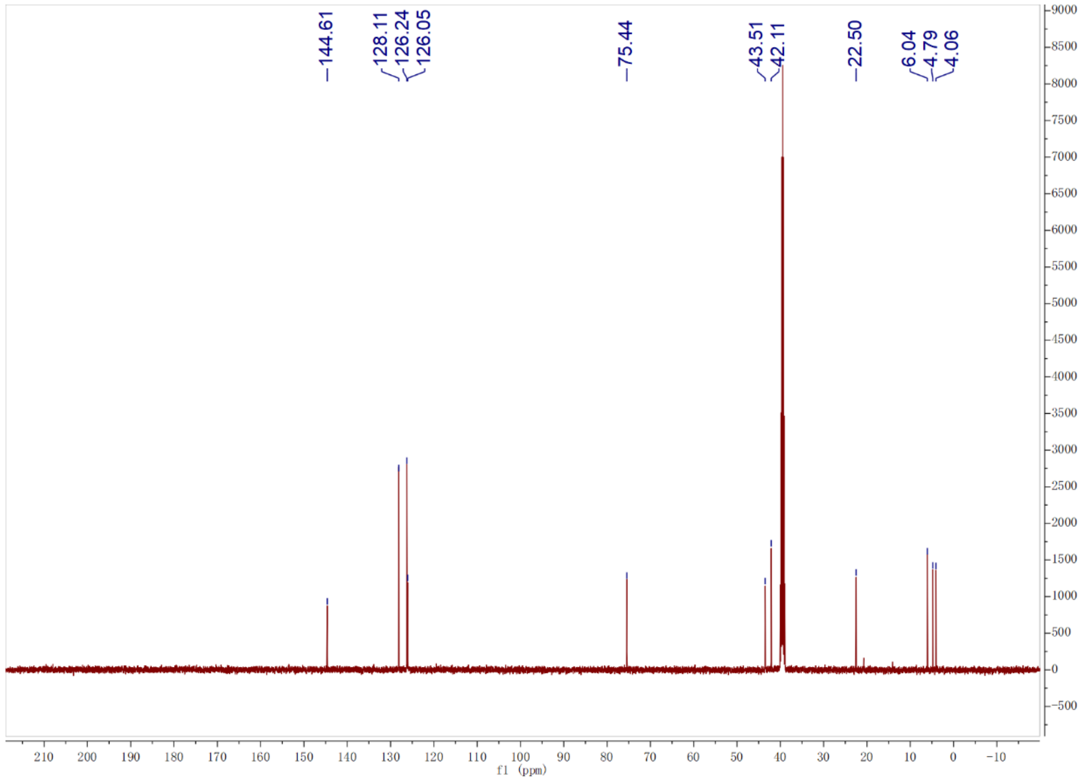

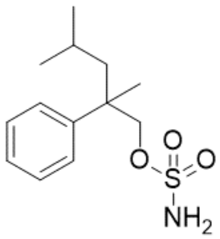

2,4-dimethyl-2-phenylpentyl sulfamate（**8**）

^{1}H NMR (500 MHz, DMSO-d6) δ 7.36 (s, 2H), 7.32 – 7.27 (m, 2H), 7.25 (t, J = 7.8 Hz, 2H), 7.17 – 7.10 (m, 1H), 4.02 – 3.91 (m, 2H), 1.59 (dd, J = 14.0, 6.1 Hz, 1H), 1.46 (dd, J = 14.0, 5.4 Hz, 1H), 1.39 – 1.32 (m, 1H), 1.28 (s, 3H), 0.66 (d, J = 6.6 Hz, 3H), 0.47 (d, J = 6.6 Hz, 3H). ^{13}C NMR (126 MHz, DMSO-d6) δ 144.17, 128.07, 126.41, 126.11, 76.46, 46.97, 41.35, 24.75, 24.28, 23.82, 22.23.; HRMS (ESI-TOF+): m/z Calcd. for $C_{13}H_{20}NO_3S$ [(M+H)+]: 270.1164. Found: 270.1165

^{1}H NMR Spectrum of 8

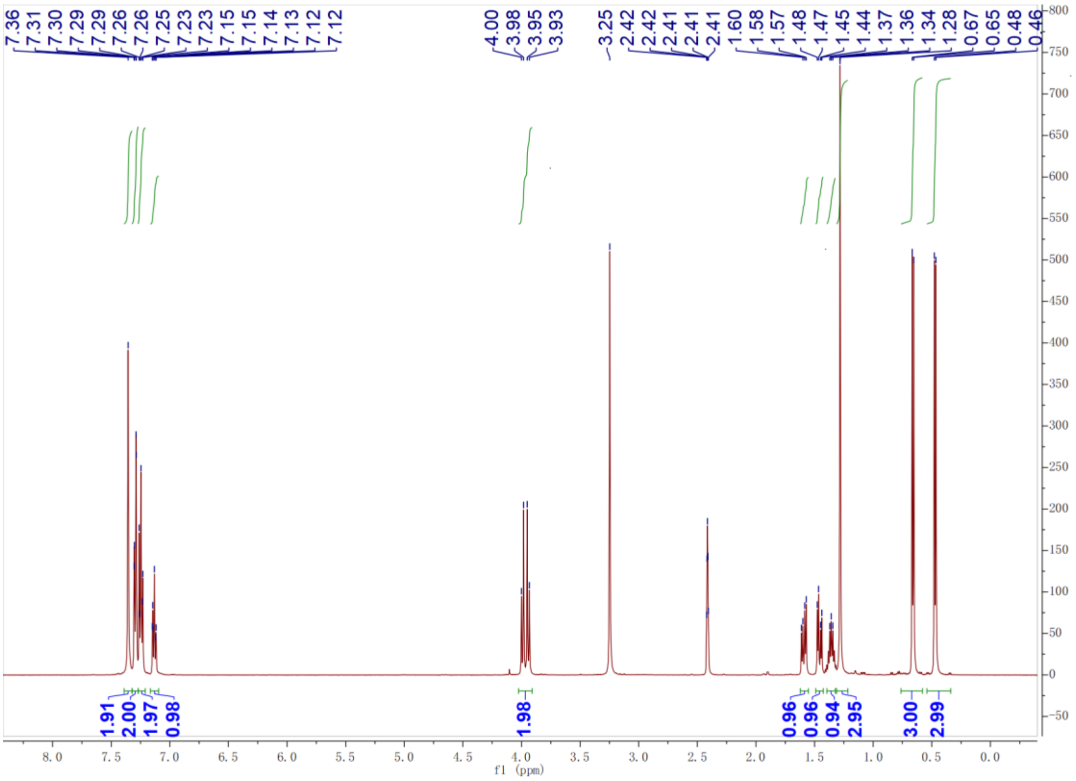

^{13}C NMR Spectrum of 8

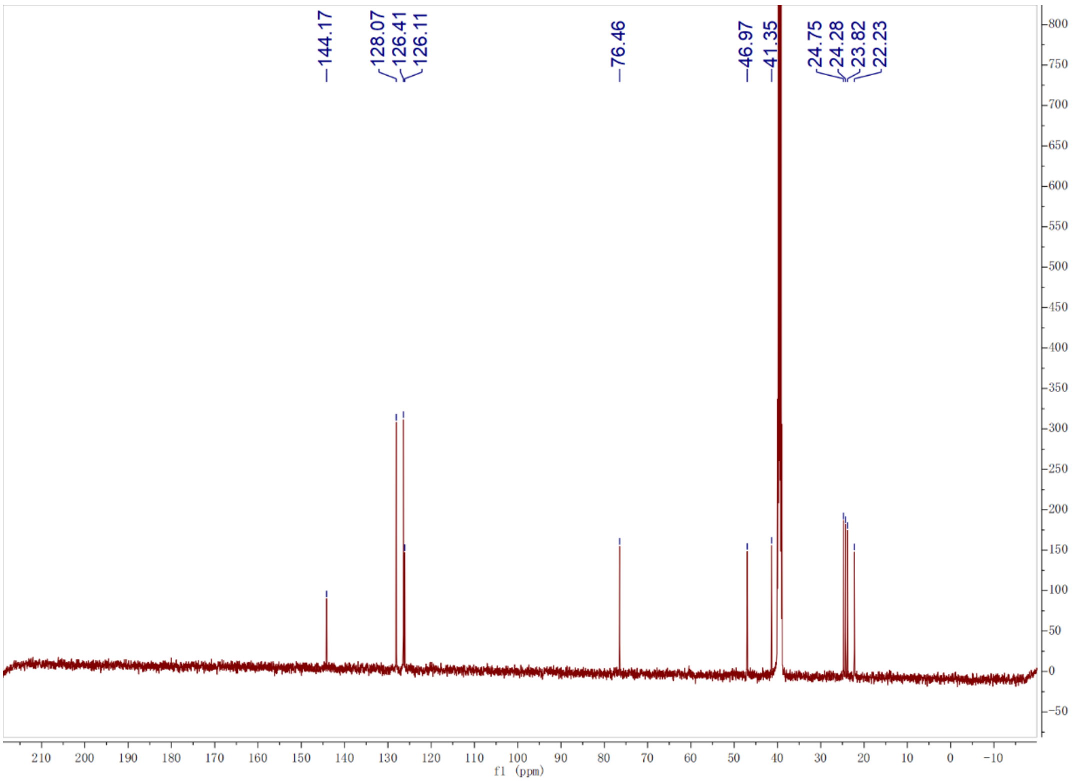

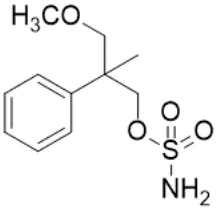

3-methoxy-2-methyl-2-phenylpropyl sulfamate (**9**)

^{1}H NMR (500 MHz, DMSO-d6) δ 7.39 (s, 2H), 7.34 – 7.28 (m, 2H), 7.25 (dd, J = 8.5, 6.8 Hz, 2H), 7.18 – 7.09 (m, 1H), 4.17 (d, J = 9.5 Hz, 1H), 4.08 (d, J = 9.5 Hz, 1H), 3.40 (s, 2H), 3.15 (s, 3H), 1.23 (s, 3H). ^{13}C NMR (126 MHz, DMSO-d6) δ 147.97, 133.38, 131.65, 82.50, 78.29, 63.91, 47.73, 25.94.; HRMS (ESI-TOF^{+}): m/z Calcd. for $C_{11}H_{18}NO_4S$ [(M+Na)$^{+}$]: 260.0957. Found: 260.0957

^{1}H NMR Spectrum of 9

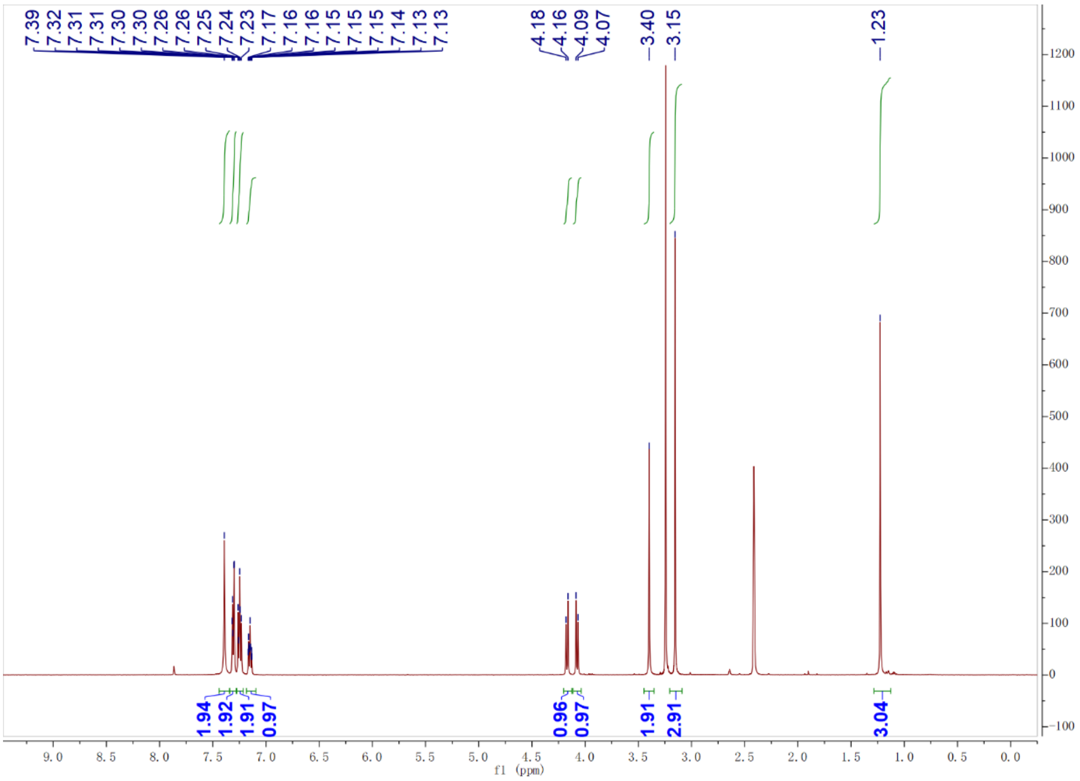

^{13}C NMR Spectrum of 9

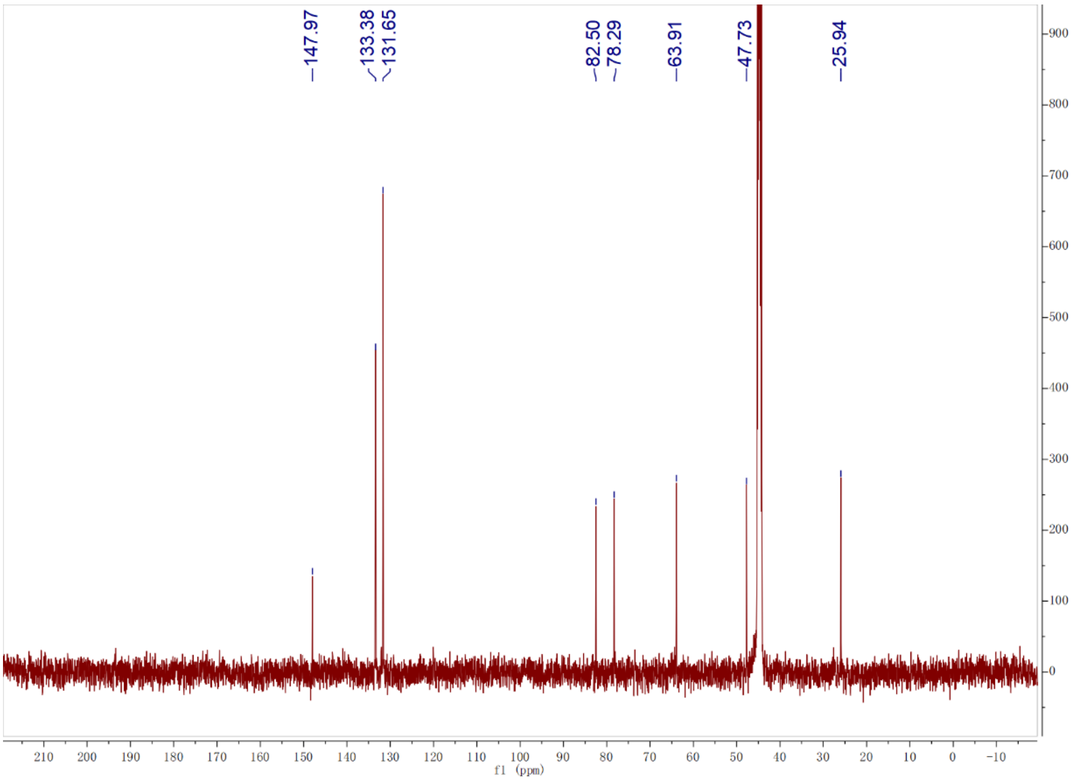

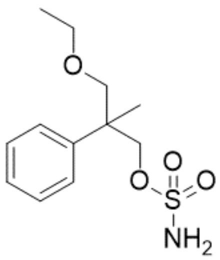

3-ethoxy-2-methyl-2-phenylpropyl sulfamate (**10**)

^{1}H NMR (500 MHz, DMSO-d6) δ 7.39 (s, 2H), 7.32 (d, J = 7.8 Hz, 2H), 7.27 – 7.23 (m, 2H), 7.15 (d, J = 7.5 Hz, 1H), 4.19 (d, J = 9.4 Hz, 1H), 4.09 (d, J = 9.4 Hz, 1H), 3.43 (s, 2H), 3.33 (dd, J = 7.0, 2.1 Hz, 2H), 1.23 (s, 3H), 0.99 (t, J = 7.0 Hz, 3H). ^{13}C NMR (126 MHz, DMSO-d6) δ 142.80, 128.11, 126.41, 126.37, 74.97, 73.12, 66.06, 42.43, 20.70, 14.87.; HRMS (ESI-TOF^{+}): m/z Calcd. for $C_{12}H_{20}NO_4S$ [(M+H)$^{+}$]: 274.1113. Found: 274.1112

^{1}H NMR Spectrum of 10

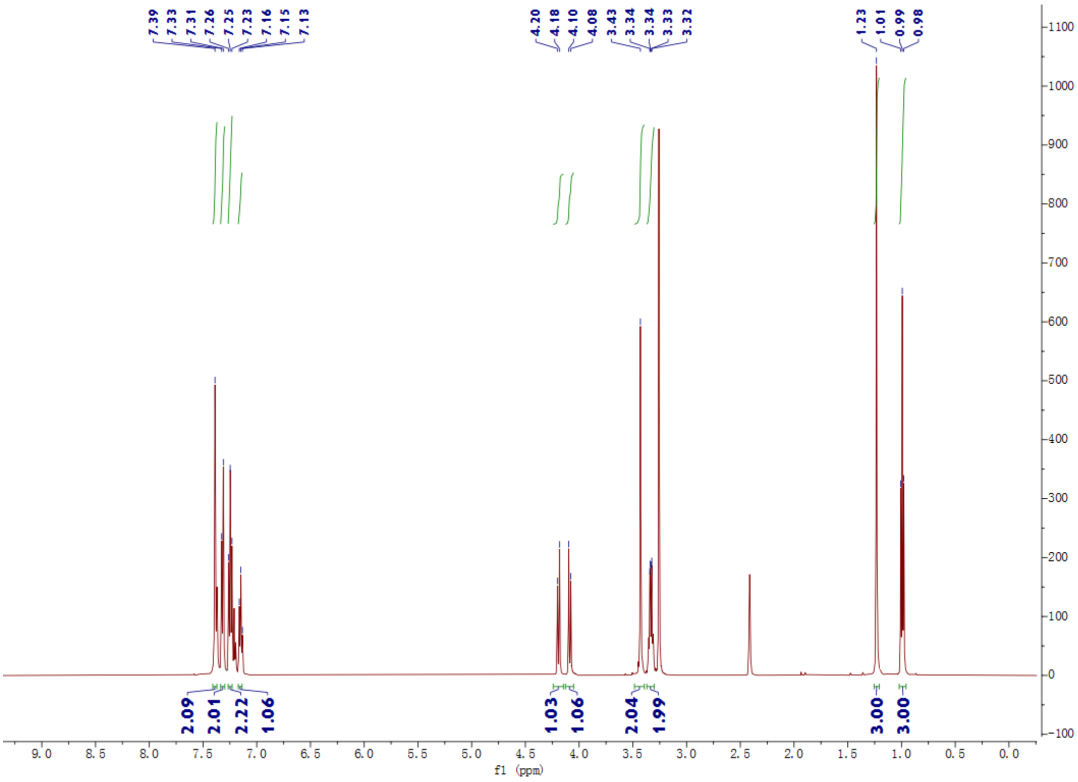

^{13}C NMR Spectrum of 10

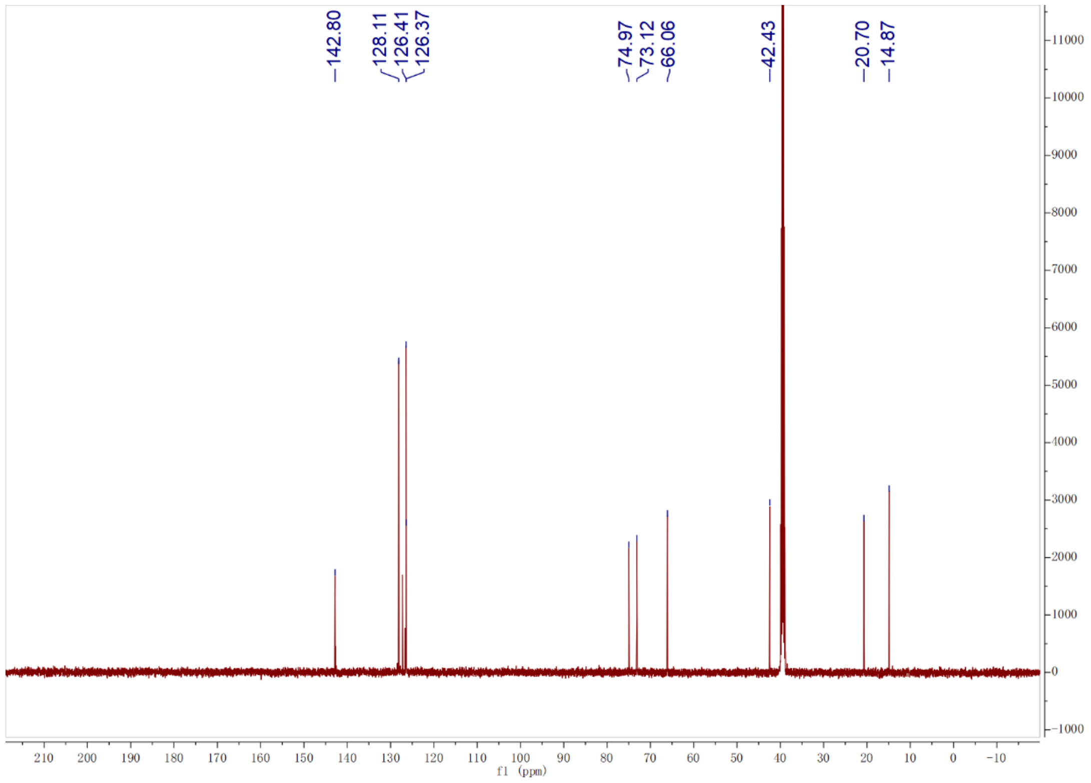

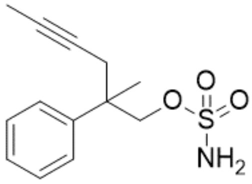

2-methyl-2-phenylhex-4-yn-1-yl sulfamate (**11**)

^{1}H NMR (500 MHz, DMSO-d_6) δ 7.41 (s, 2H), 7.34 (d, *J* = 7.5 Hz, 2H), 7.26 (t, *J* = 7.8 Hz, 2H), 7.15 (t, *J* = 7.2 Hz, 1H), 4.15 (d, *J* = 9.4 Hz, 1H), 4.06 (d, *J* = 9.4 Hz, 1H), 2.51 – 2.40 (m, 2H), 1.60 (t, *J* = 2.6 Hz, 3H), 1.31 (s, 3H). ^{13}C NMR (500 MHz, DMSO-d6) δ 143.42, 128.12, 126.41, 126.26, 78.21, 75.68, 74.66, 41.09, 28.34, 22.59, 3.14.; HRMS (ESI-TOF$^+$): m/z Calcd. for $C_{13}H_{18}NO_3S$ [(M+H)$^+$]: 268.1007. Found: 268.1007

^{1}H NMR Spectrum of 11

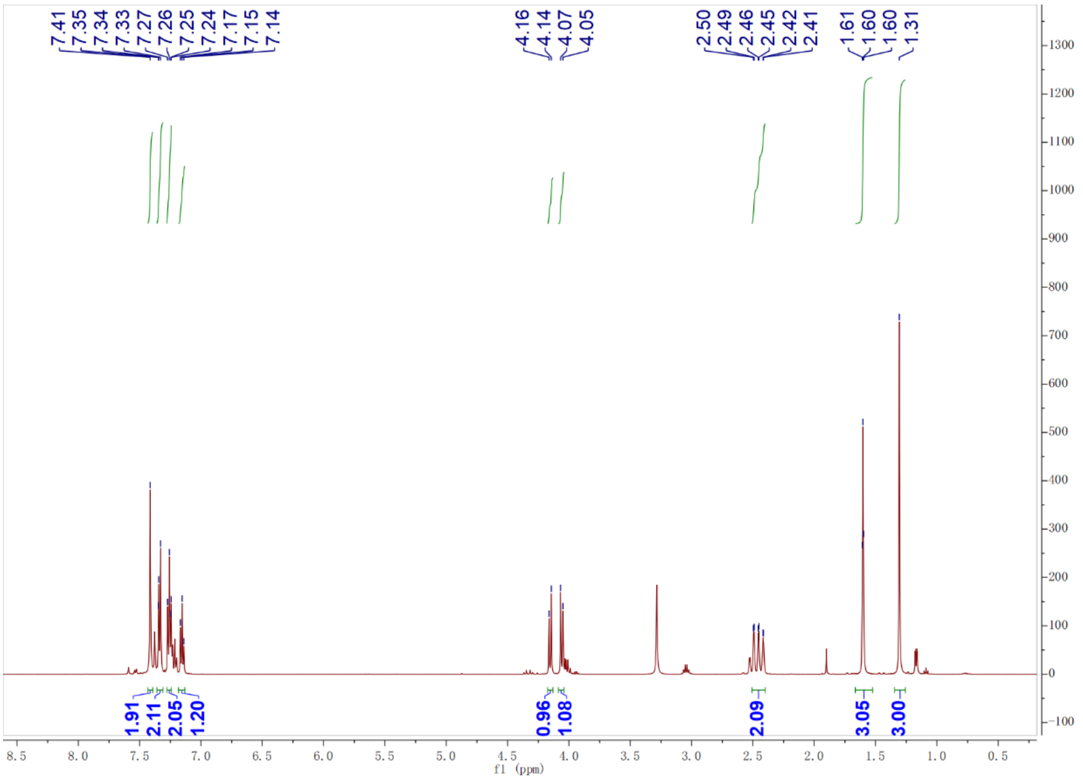

^{13}C NMR Spectrum of 11

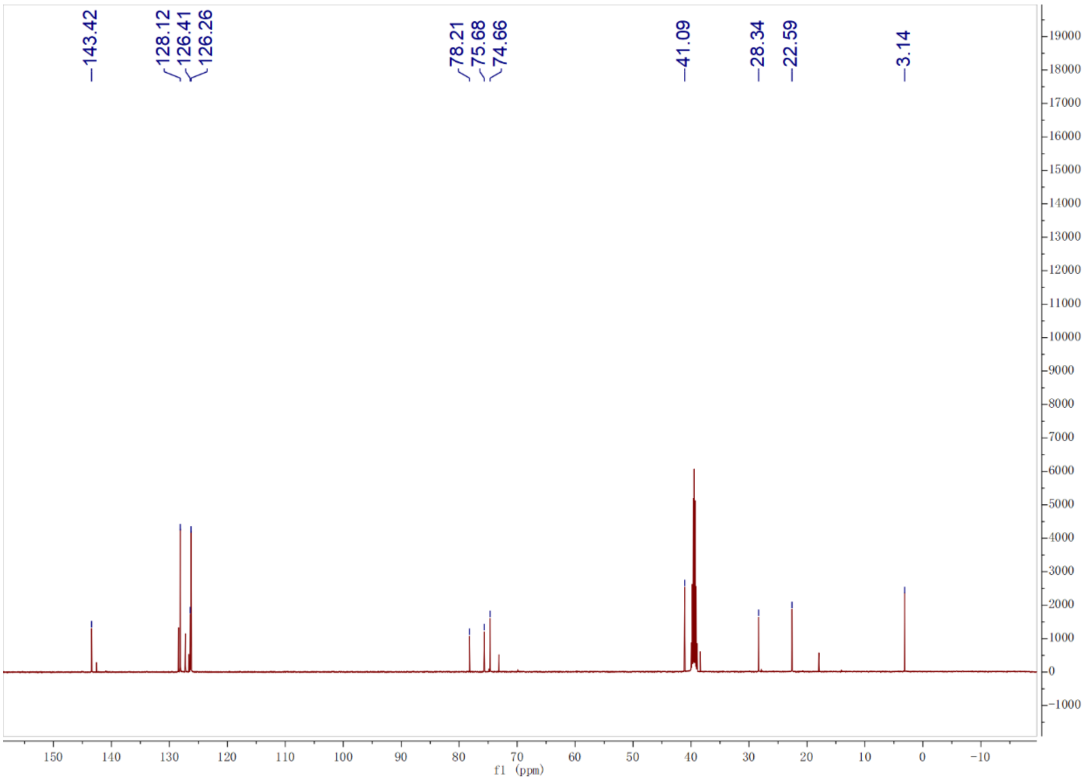

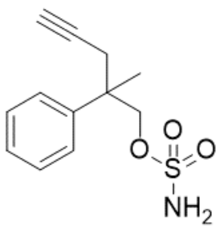

2-methyl-2-phenylpent-4-yn-1-yl sulfamate (**12**)

^{1}H NMR (500 MHz, DMSO-d6) δ 7.42 (s, 2H), 7.38 – 7.31 (m, 2H), 7.26 (t, J = 7.8 Hz, 2H), 7.19 – 7.12 (m, 1H), 4.14 (d, J = 9.5 Hz, 1H), 4.06 (d, J = 9.6 Hz, 1H), 2.72 (t, J = 2.6 Hz, 1H), 2.59 (dd, J = 16.9, 2.7 Hz, 1H), 2.48 (dd, J = 16.8, 2.7 Hz, 1H), 1.32 (s, 3H). ^{13}C NMR (126 MHz, DMSO-d6) δ 148.28, 133.40, 131.77, 131.55, 86.22, 79.80, 78.83, 46.23, 33.10, 27.64.; HRMS (ESI-TOF$^+$): m/z Calcd. for $C_{12}H_{16}NO_3S$ $[(M+H)^+]$: 254.0851. Found: 254.0852

^{1}H NMR Spectrum of 12

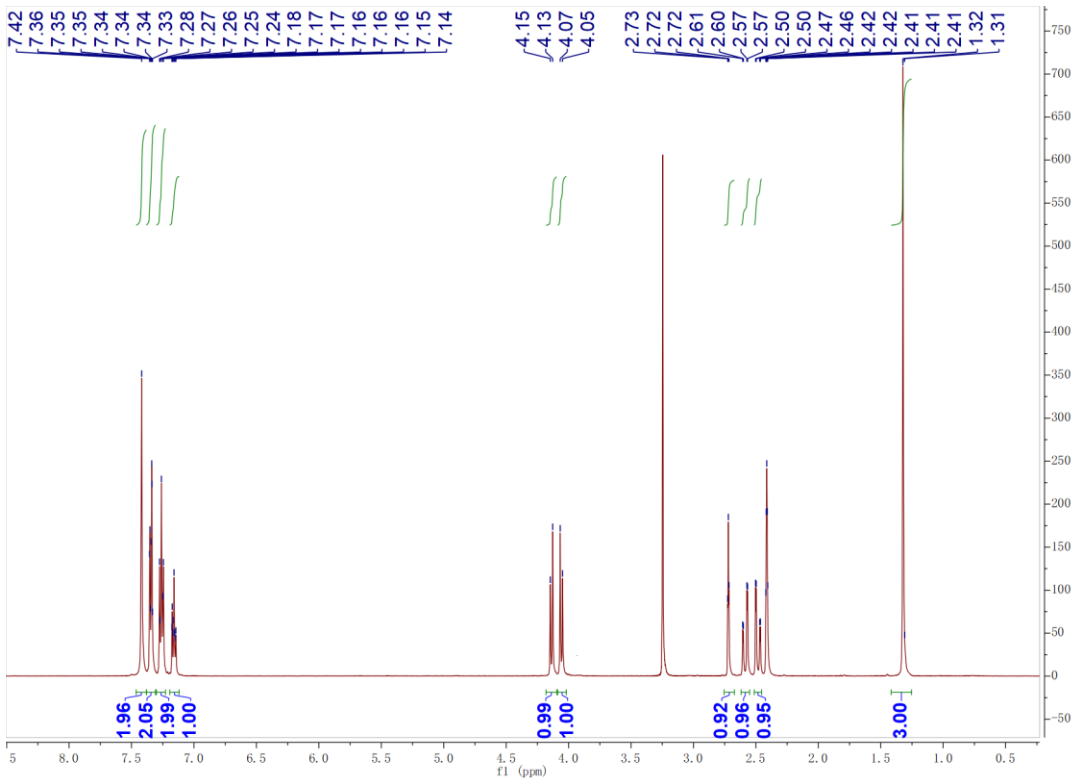

^{13}C NMR Spectrum of 12

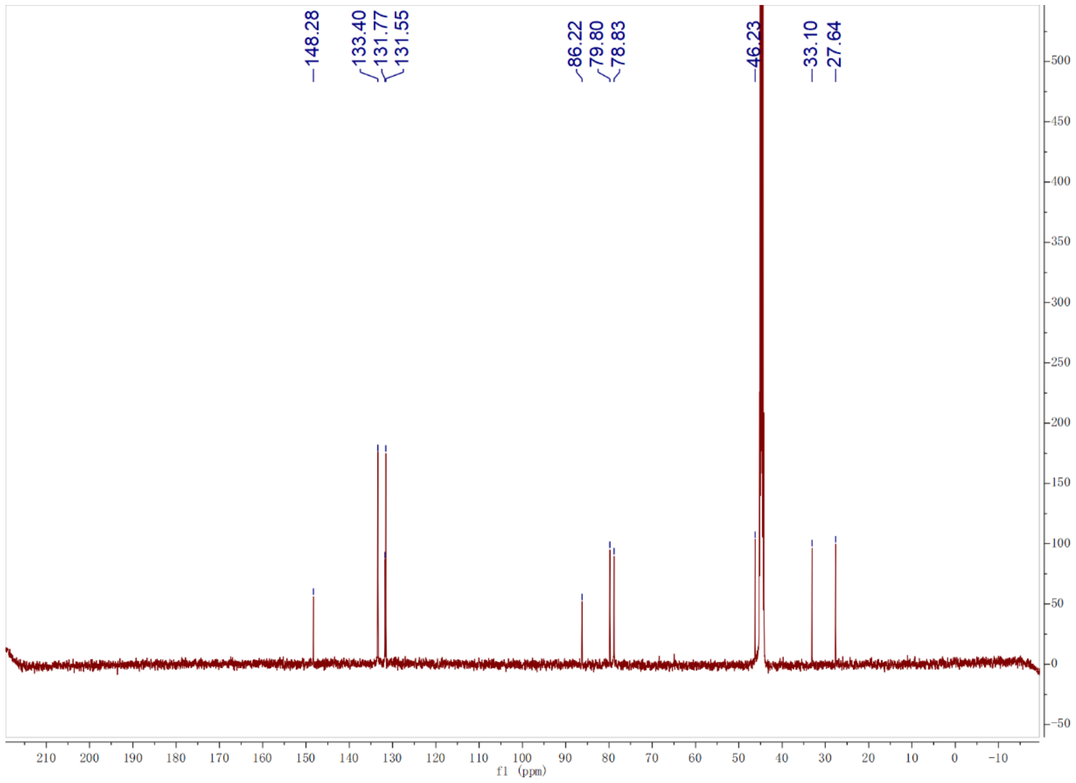

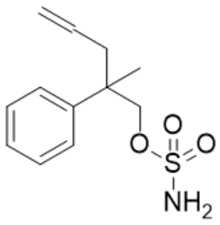

2-methyl-2-phenylpent-4-en-1-yl sulfamate (**13**)

^{1}H NMR (500 MHz, DMSO-d6) δ 7.39 (s, 2H), 7.32 – 7.28 (m, 2H), 7.27 – 7.24 (m, 2H), 7.14 (t, J = 7.1 Hz, 1H), 5.39 (ddt, J = 17.2, 10.1, 7.3 Hz, 1H), 5.01 – 4.84 (m, 2H), 4.05 (d, J = 9.5 Hz, 1H), 4.00 (d, J = 9.5 Hz, 1H), 2.46 – 2.42 (m, 1H), 2.29 (dd, J = 13.9, 7.6 Hz, 1H), 1.23 (s, 3H). ^{13}C NMR (126 MHz, DMSO-d6) δ 143.74, 133.73, 128.18, 126.31, 126.23, 118.23, 75.44, 42.63, 41.01, 22.04.; HRMS (ESI-TOF$^+$): m/z Calcd. for $C_{12}H_{18}NO_3S$ [(M+H)$^+$]: 256.1007. Found: 256.1007

^{1}H NMR Spectrum of 13

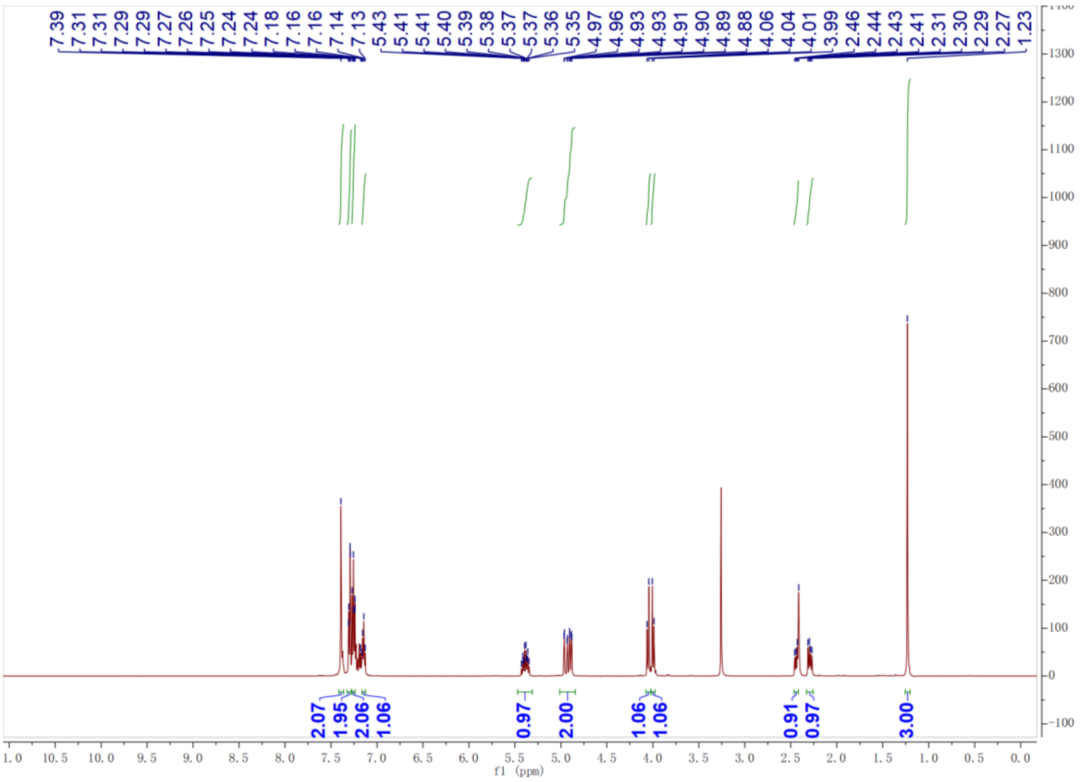

^{13}C NMR Spectrum of 13

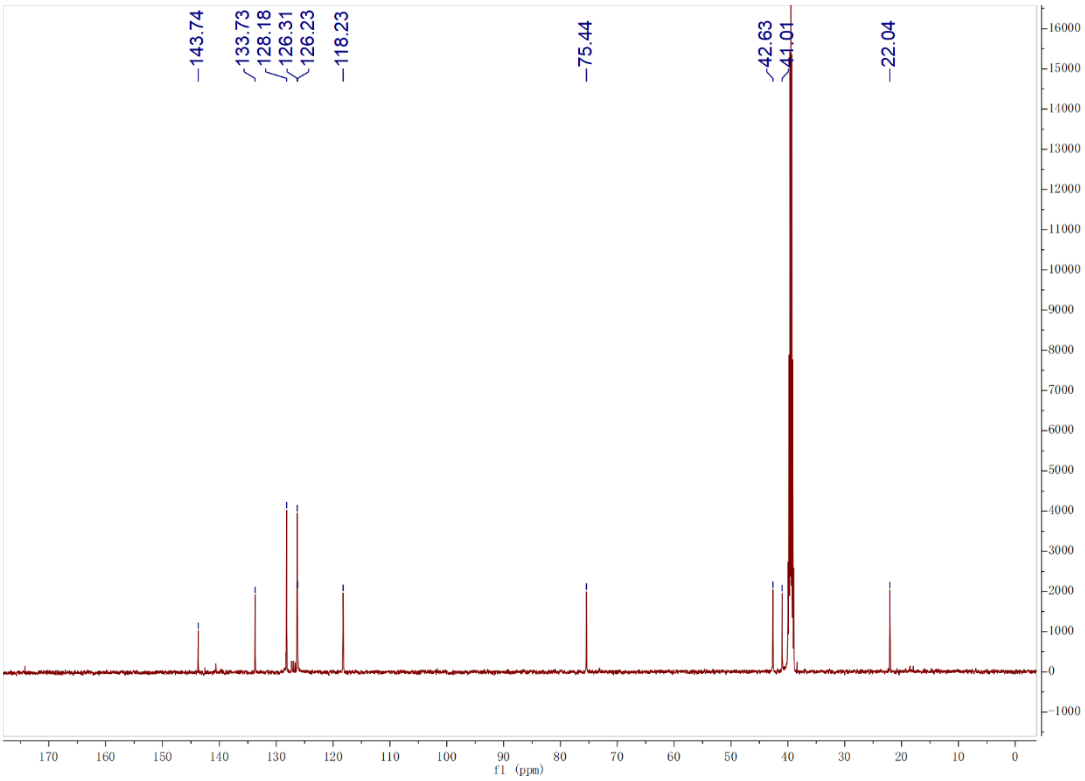

Characterization data and spectra of compound 1a-12a

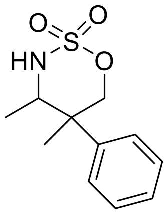

4,5-dimethyl-5-phenyl-1,2,3-oxathiazinane 2,2-dioxide（**1a**）

76% yield, dr=5.1:1, yellow oil. Major product: ^{1}H NMR (500 MHz, DMSO-d6) δ 7.77 (d, *J* = 9.8 Hz, 1H), 7.43 -7.30 (m, 5H), 4.63 (dd, *J* = 11.4, 0.9 Hz, 1H), 4.11 (d, *J* = 11.4 Hz, 1H), 4.10-4.02 (m, 1H), 1.43 (d, *J* = 0.8 Hz, 3H), 0.76 (d, *J* = 6.8 Hz, 3H). ^{13}C NMR (101 MHz, DMSO-d6) δ 140.46, 129.25, 127.79, 127.16, 81.00, 58.11, 40.01, 14.93, 13.86.; Minor product: ^{1}H NMR (400 MHz, DMSO-d6) δ 7.61 (d, *J* = 8.3 Hz, 1H), 7.52-7.45 (m, 2H), 7.29 (m, 3H), 4.75 (d, *J* = 12.0 Hz, 1H), 4.54 (d, *J* = 12.0 Hz, 1H), 4.06-4.03 (m, 1H), 3.75-3.69 (m, 1H), 1.28 (s, 2H), 0.92 (d, *J* = 6.9 Hz, 3H). ^{13}C NMR (101 MHz, DMSO-d6) δ 140.93, 128.51, 128.21, 127.26, 79.81, 59.09, 38.60, 22.26, 16.29.; HRMS (ESI-TOF$^+$): m/z Calcd. for $C_{11}H_{15}NNaO_3S$ [(M+Na)$^+$]: 264.0670. Found: 264.0678.

^{1}H NMR Spectrum of 1a

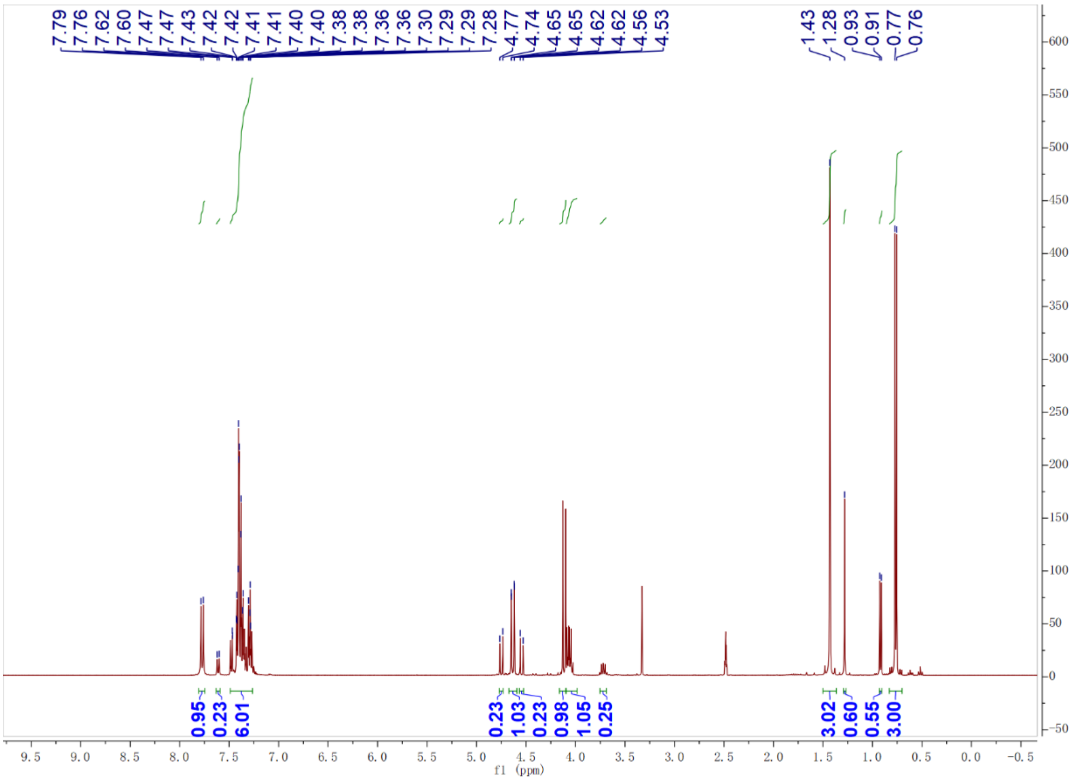

^{13}C NMR Spectrum of 1a

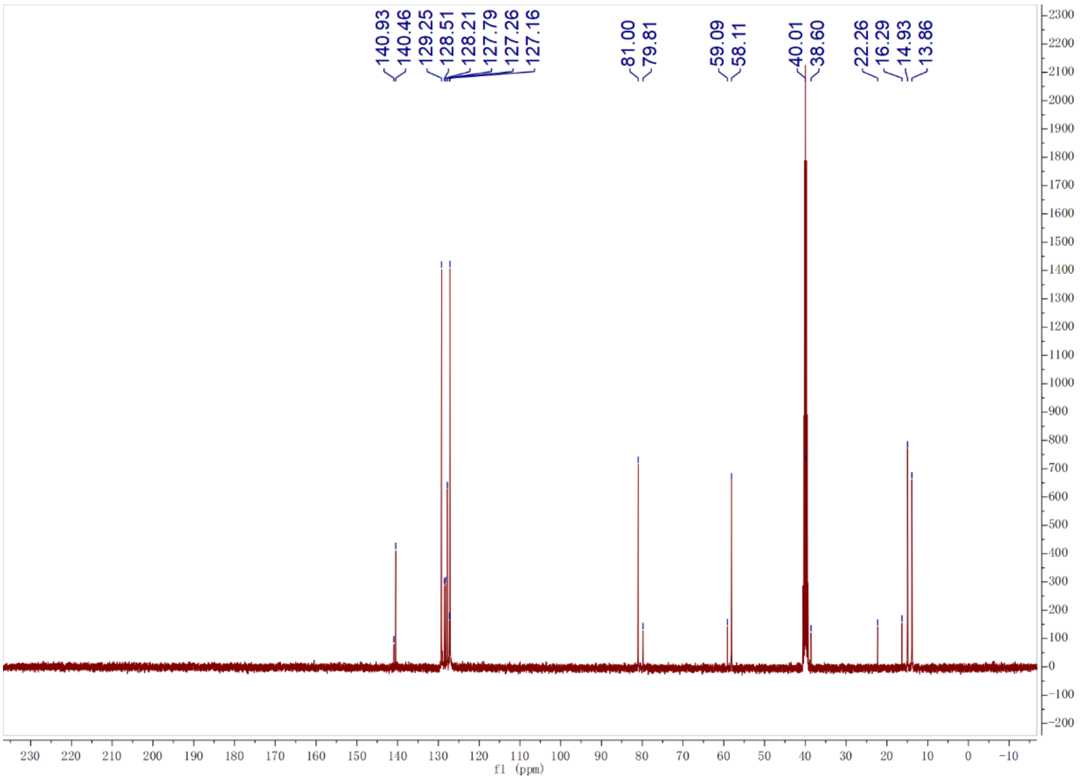

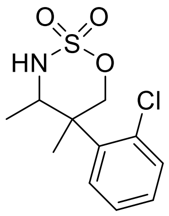

5-(2-chlorophenyl)-4,5-dimethyl-1,2,3-oxathiazinane 2,2-dioxide（**2a**）

70% yield, dr>20:1, yellow oil. ^{1}H NMR (500 MHz, DMSO-d6) δ 7.56 (dd, J = 7.1, 2.2 Hz, 1H), 7.50 (dd, J = 7.3, 2.2 Hz, 1H), 7.40 (ddd, J = 6.9, 4.6, 2.0 Hz, 3H), 5.17 (d, J = 13.1 Hz, 1H), 4.80 (d, J = 13.1 Hz, 1H), 3.92 (t, J = 5.5 Hz, 1H), 1.67 (s, 3H), 1.23 (d, J = 10.3 Hz, 3H) ^{13}C NMR (126 MHz, DMSO-d6) δ 139.78, 132.52, 131.84, 129.29, 128.09, 127.45, 74.44, 54.47, 36.30, 34.94, 22.82. ; HRMS (ESI-TOF^{+}): m/z Calcd. for $C_{11}H_{14}ClNNaO_3S$ [(M+Na)$^{+}$]: 298.0281. Found: 298.0283.

^{1}H NMR Spectrum of 2a

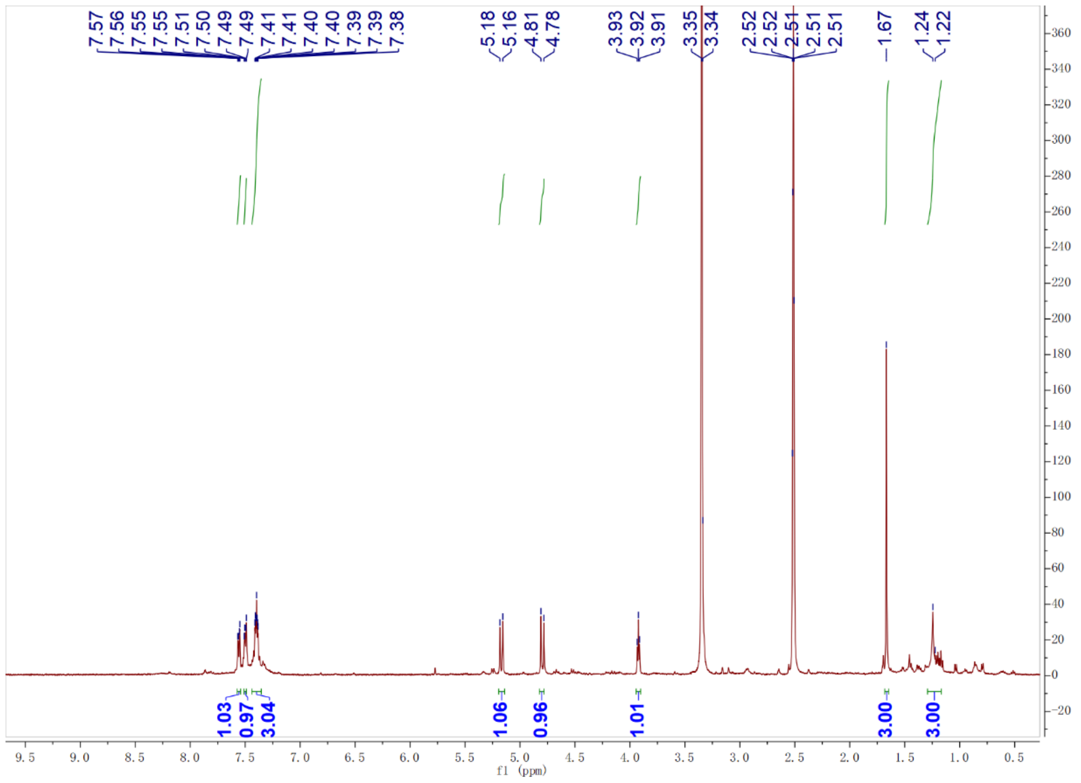

^{13}C NMR Spectrum of 2a

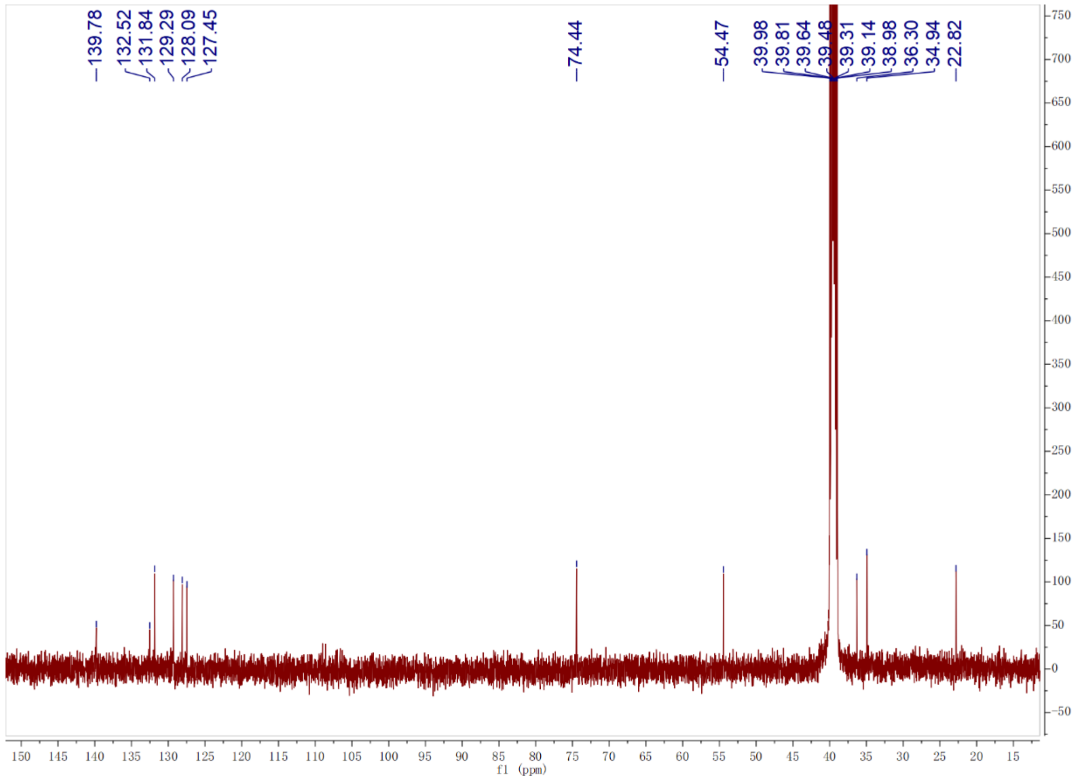

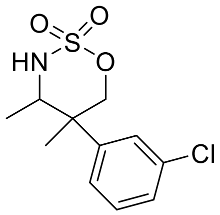

5-(3-chlorophenyl)-4,5-dimethyl-1,2,3-oxathiazinane 2,2-dioxide(**3a**)

78% yield, dr=2:1, yellow oil. Major product: ^{1}H NMR (500 MHz, DMSO-d6) δ 7.84 (d, J = 9.8 Hz, 1H), 7.49 -7.39 (m, 5H), 4.63 (d, J = 11.4 Hz, 1H), 4.19 (d, J = 11.5 Hz, 1H), 4.13-4.05 (m, 1H), 1.45 (s, 3H), 0.81 (d, J = 6.8 Hz, 3H). ^{13}C NMR (126 MHz, DMSO-d6) δ 142.72, 133.57, 130.56, 127.44, 126.90, 125.51, 80.09, 57.38, 39.47.14.50, 13.38.; Minor product: ^{1}H NMR (500 MHz, DMSO-d6) δ 7.68 (d, J = 8.7 Hz, 1H), 7.58 (d, J = 1.9 Hz, 1H), 7.42-7.36 (m, 3H), 4.78 (d, J = 12.2 Hz, 1H), 4.57 (d, J = 12.1 Hz, 1H), 4.10-4.04 (m, 1H), 3.76 (dd, J = 8.4, 6.6 Hz, 1H), 1.30 (s, 2H), 0.96 (d, J = 6.9 Hz, 3H). ^{13}C NMR (126 MHz, DMSO-d6) δ 132.88, 129.64, 127.98, 126.86, 79.31, 58.48, 38.34, 21.27, 15.65.; HRMS (ESI-TOF+): m/z Calcd. for $C_{11}H_{14}ClNNaO_3S$ [(M+Na)$^{+}$]: 298.0281. Found: 298.0284.

^{1}H NMR Spectrum of 3a

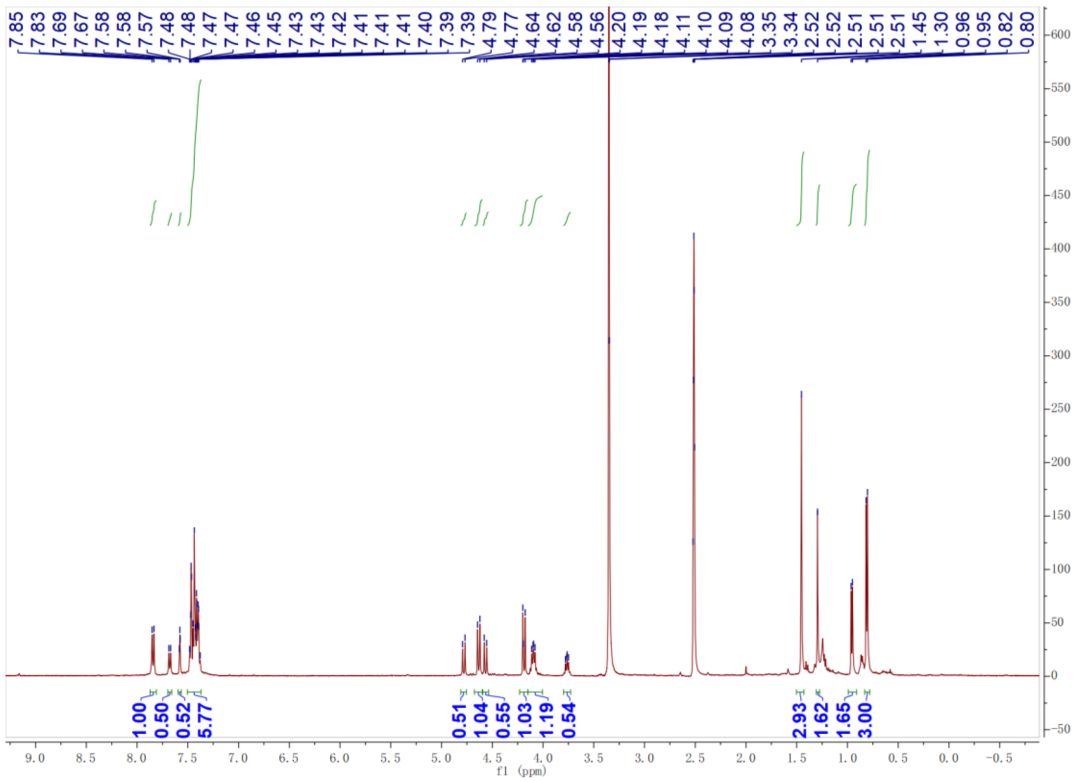

^{13}C NMR Spectrum of 3a

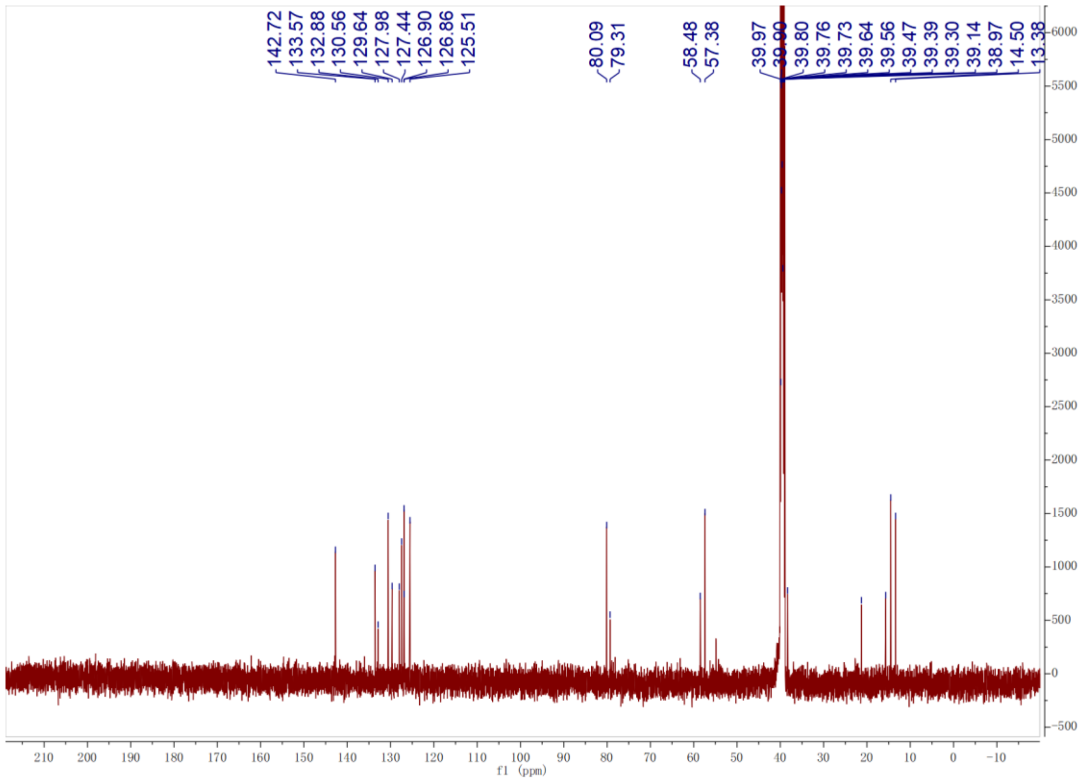

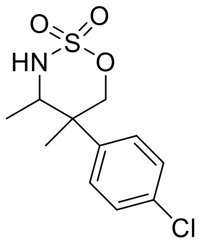

5-(4-chlorophenyl)-4,5-dimethyl-1,2,3-oxathiazinane 2,2-dioxide(**4a**)

80% yield, dr=3.8:1, yellow oil. Major product: ^{1}H NMR (500 MHz, DMSO-d6) δ 7.80 (d, *J* = 9.8 Hz, 1H), 7.45-7.41 (m, 4H), 4.63-4.57 (m, 1H), 4.12 (d, *J* = 11.4 Hz, 1H), 4.07-4.00 (m, 1H), 1.45-1.37 (m, 3H), 0.76 (d, *J* = 6.8 Hz, 3H). ^{13}C NMR (101 MHz, DMSO-d6) δ 139.52, 132.57, 129.30, 129.13, 80.64, 57.97, 39.89, 14.91, 13.84.; Minor product: ^{1}H NMR (400 MHz, DMSO-d6) δ 7.56 (d, J = 8.7 Hz, 1H), 7.53-7.49 (m, 2H), 7.43-7.39 (m, 2H), 4.72 (d, *J* = 12.0 Hz, 1H), 4.53 (d, *J* = 12.1 Hz, 1H), 4.03 (dq, *J* = 9.9, 6.8 Hz, 1H), 3.72 (dd, *J* = 8.7, 6.9 Hz, 1H), 1.25 (s, 2H), 0.91 (d, *J* = 6.9 Hz, 3H). ^{13}C NMR (101 MHz, DMSO-d6) δ 139.64, 132.14, 130.46, 79.90, 58.99, 16.14.; HRMS (ESI-TOF^{+}): m/z Calcd. for $C_{11}H_{14}ClNNaO_3S$ [(M+Na)$^{+}$]: 298.0281. Found: 298.0281.

^{1}H NMR Spectrum of 4a

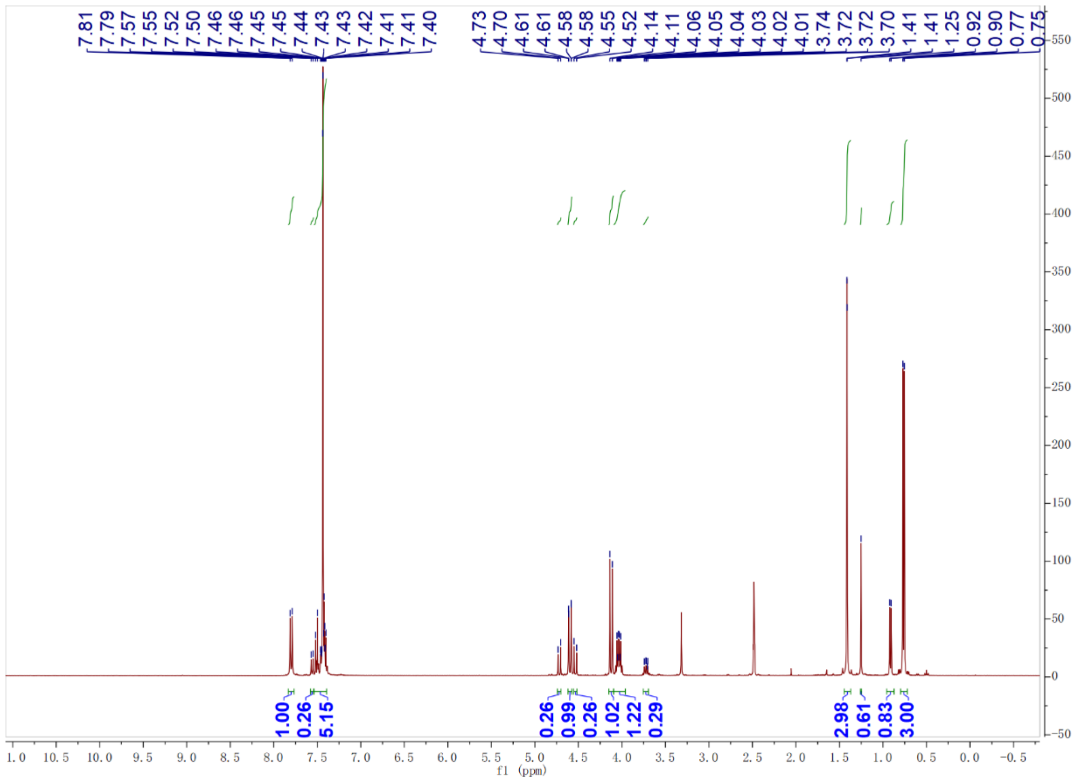

. ^{13}C NMR Spectrum of 4a

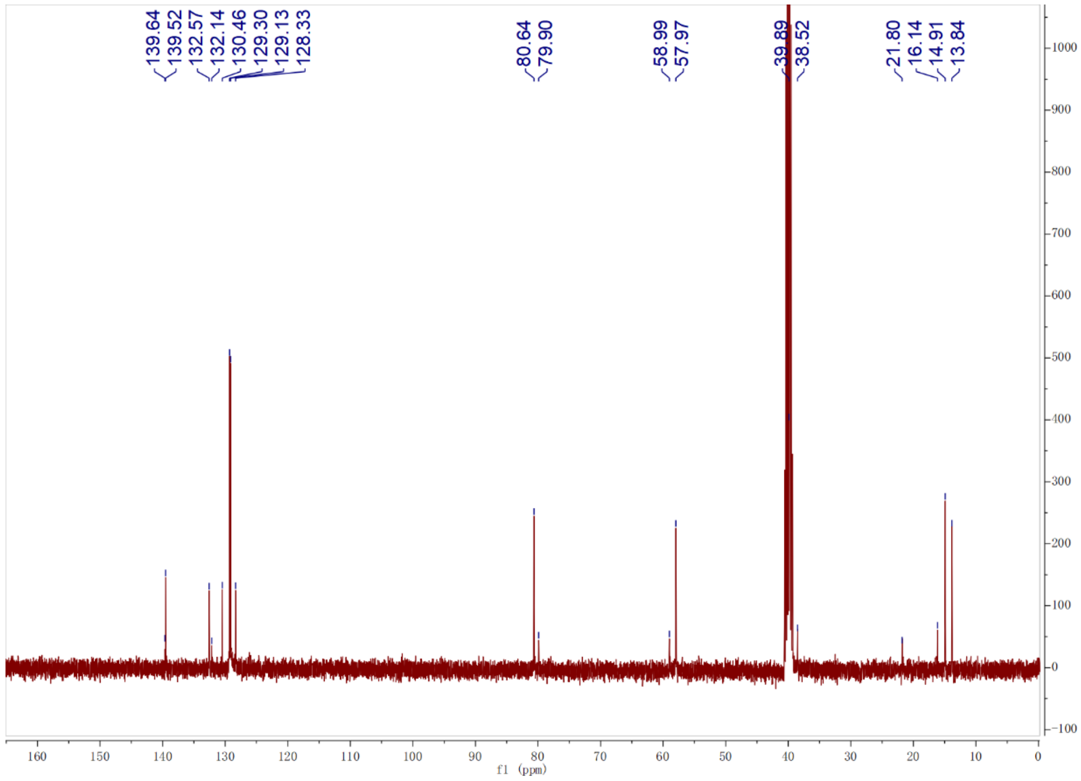

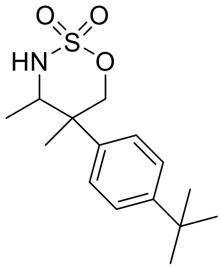

5-(4-(tert-butyl)phenyl)-4,5-dimethyl-1,2,3-oxathiazinane 2,2-dioxide（**5a**）

50% yield, dr=1.8:1, yellow oil. Major product: ^{1}H NMR (500 MHz, DMSO-d6) δ 7.77 (d, *J* = 9.8 Hz, 1H), 7.45-7.34 (m, 5H), 4.63 (d, *J* = 11.5 Hz, 1H), 4.13 (d, *J* = 11.4 Hz, 1H), 4.07 (dd, *J* = 9.8, 6.7 Hz, 1H), 1.44 (s, 3H), 1.27 (s, 9H), 0.79 (d, *J* = 6.8 Hz, 3H). ^{13}C NMR (126 MHz, DMSO-d6) δ 149.56, 136.92, 126.96, 126.33, 125.50, 57.57, 37.86, 31.00, 14.53, 13.43.; Minor product: ^{1}H NMR (500 MHz, DMSO-d6) δ 7.59 (d, *J* = 8.4 Hz, 1H), 7.39-7.33 (m, 4H), 4.75 (d, *J* = 11.9 Hz, 1H), 4.54 (d, *J* = 11.9 Hz, 1H), 4.07 (dd, *J* = 9.8, 6.7 Hz, 1H), 3.72 (dd, *J* = 8.2, 6.7 Hz, 1H), 1.29 (s, 9H), 1.25 (s, 2H), 0.96 (d, *J* = 6.9 Hz, 3H). ^{13}C NMR (126 MHz, DMSO-d6) δ 148.84, 137.39, 127.47, 124.75, 79.49, 58.66, 34.07, 21.77, 15.81, 14.53, 13.43.; HRMS (ESI-TOF$^+$): m/z Calcd. for $C_{15}H_{23}NNaO_4S$ [(M+Na)$^+$]: 320.1296. Found: 320.1293.

^{1}H NMR Spectrum of 5a

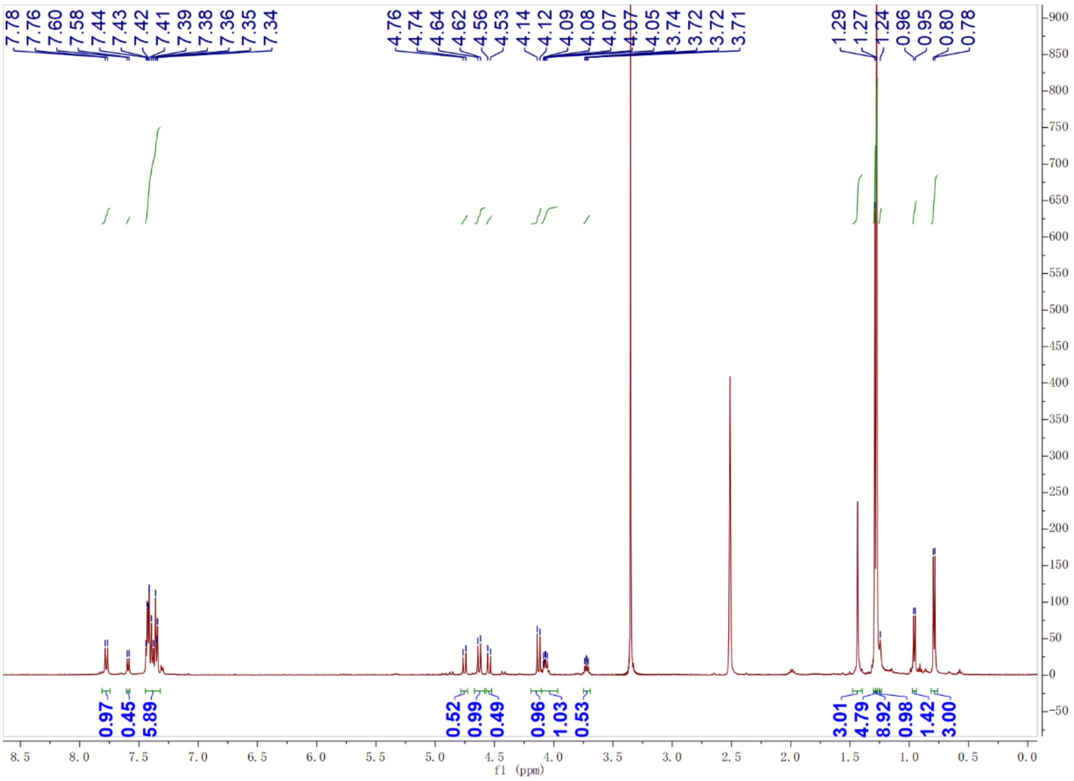

^{13}C NMR Spectrum of 5a

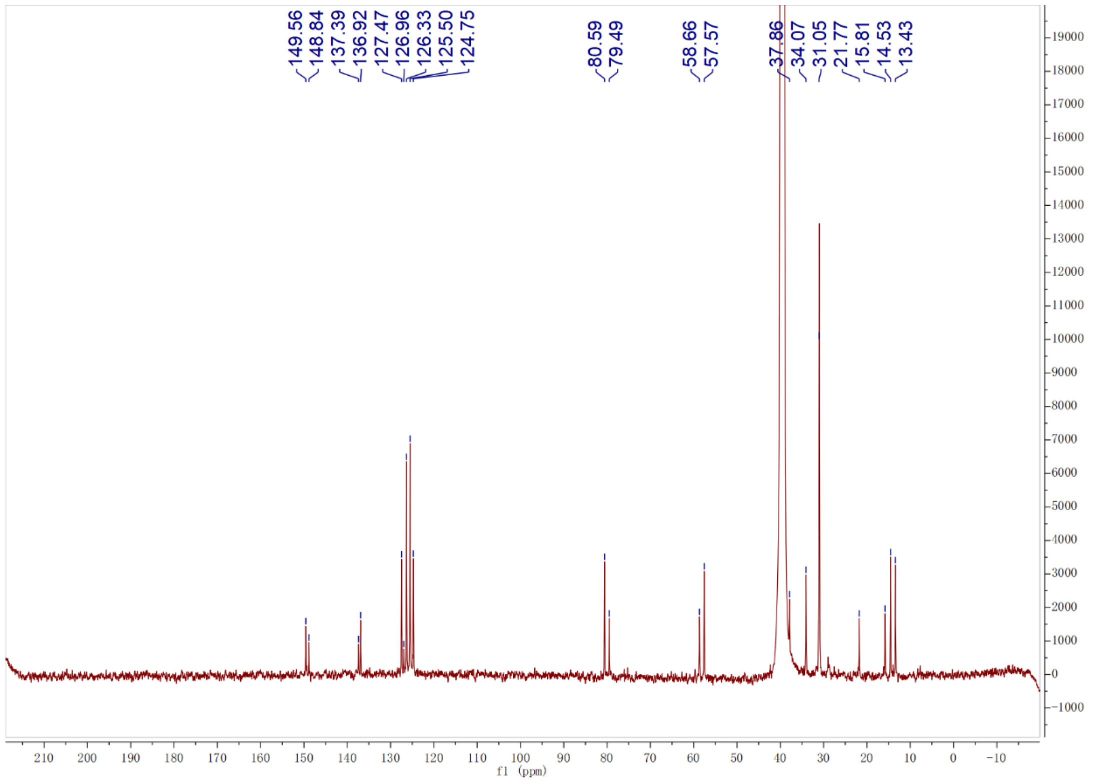

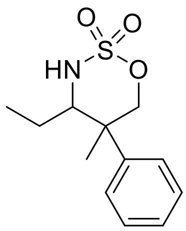

4-ethyl-5-methyl-5-phenyl-1,2,3-oxathiazinane 2,2-dioxide(**6a)**

70% yield, dr>20:1, yellow oil. ^{1}H NMR (400 MHz, DMSO-d6) δ 7.68 (d, J = 10.1 Hz, 1H), 7.43 – 7.35 (m, 4H), 7.31 – 7.26 (m, 1H), 4.61 (dd, J = 11.4, 0.8 Hz, 1H), 4.08 (d, J = 11.4 Hz, 1H), 3.78 (td, J = 10.6, 2.4 Hz, 1H), 1.42 (s, 3H), 1.28 – 1.19 (m, 1H), 0.97 – 0.89 (m, 1H), 0.75 (t, J = 7.3 Hz, 3H). ^{13}C NMR (400 MHz, DMSO-d6) δ 140.58, 129.28, 127.78, 127.16, 81.00, 64.78, 21.76, 14.48, 11.14.; HRMS (ESI-TOF^{+}): m/z Calcd. for $C_{12}H_{17}NNaO_3S$ [(M+H)$^{+}$]: 256.1007. Found: 256.1026

^{1}H NMR Spectrum of 6a

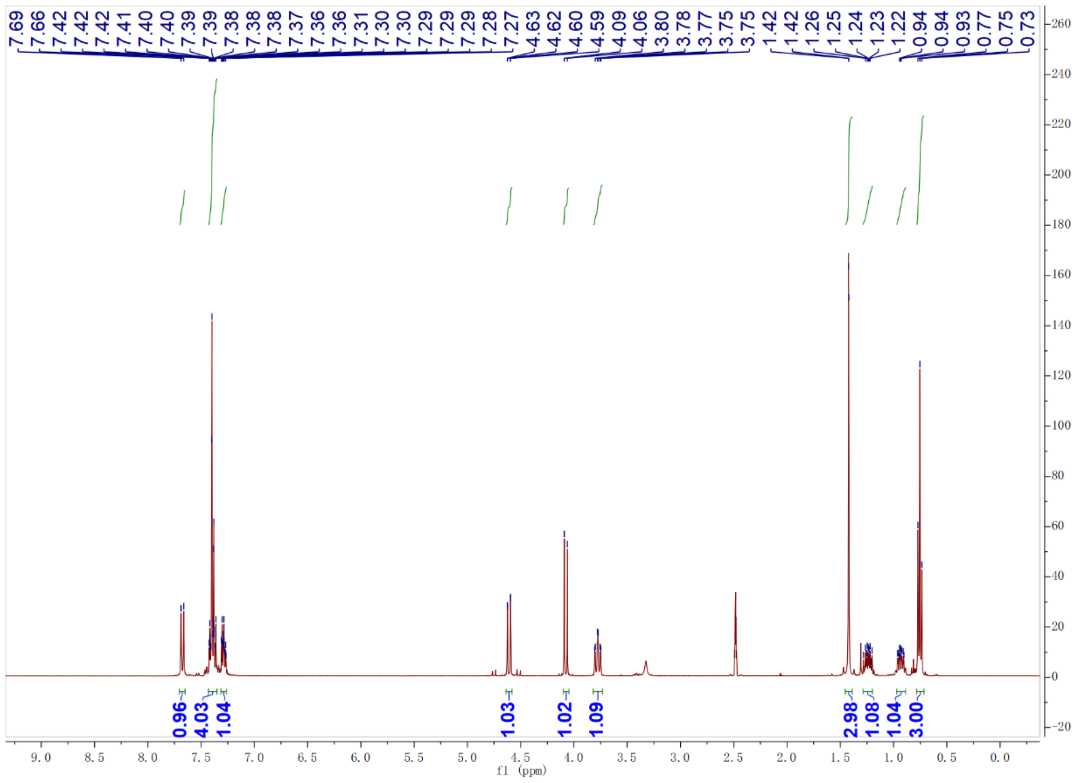

^{13}C NMR Spectrum of 6a

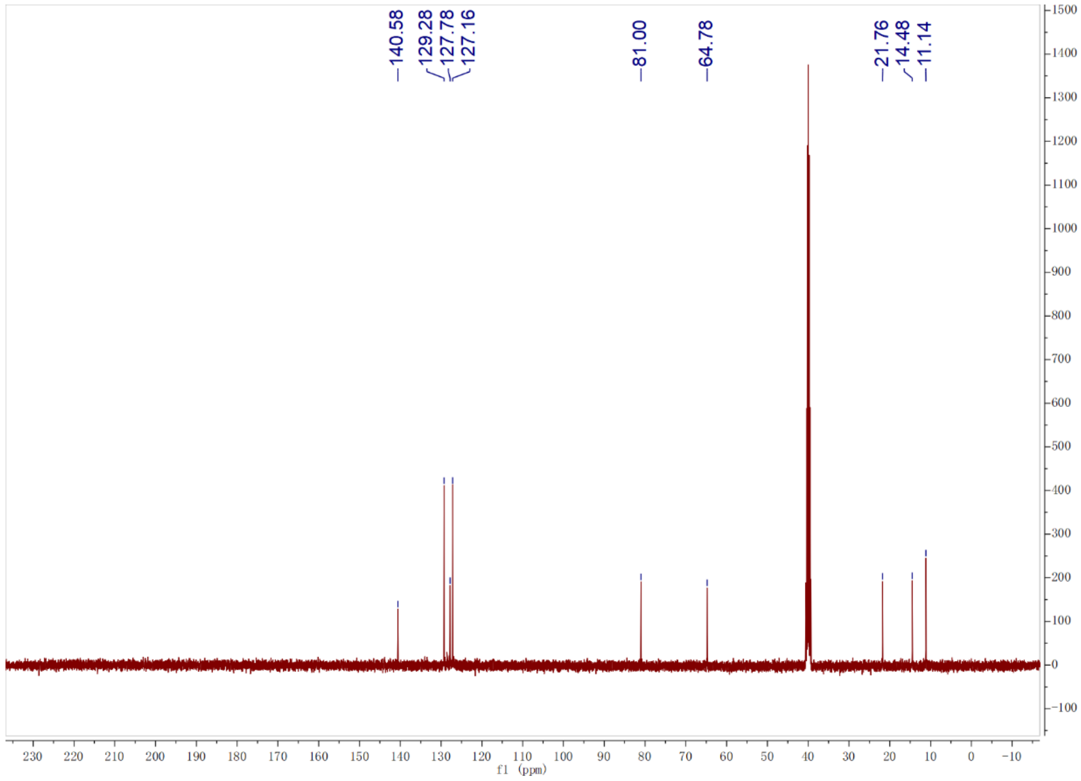

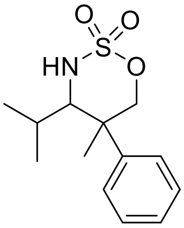

4-isopropyl-5-methyl-5-phenyl-1,2,3-oxathiazinane 2,2-dioxide（**7a**）

75% yield, dr>20:1, yellow oil. ^{1}H NMR (500 MHz, Chloroform-d) δ 7.48 – 7.40 (m, 4H), 7.38 – 7.33 (m, 1H), 4.86 (d, J = 11.7 Hz, 1H), 4.38 (s, 1H), 4.08 (dd, J = 11.3, 4.8 Hz, 1H), 3.93 (d, J = 11.8 Hz, 1H), 1.63 (s, 3H), 1.29 (s, 1H), 0.97 (d, J = 6.7 Hz, 3H), 0.66 (d, J = 6.8 Hz, 3H). ^{13}C NMR (126 MHz, Chloroform-d) δ 139.73, 129.04, 127.86, 126.66, 81.94, 67.16, 40.71, 28.95, 22.33, 18.65, 14.74.; HRMS (ESI-TOF^{+}): m/z Calcd. for $C_{13}H_{19}NNaO_3S$ [(M+H)$^{+}$]: 270.1164. Found: 270.1166

^{1}H NMR Spectrum of 7a

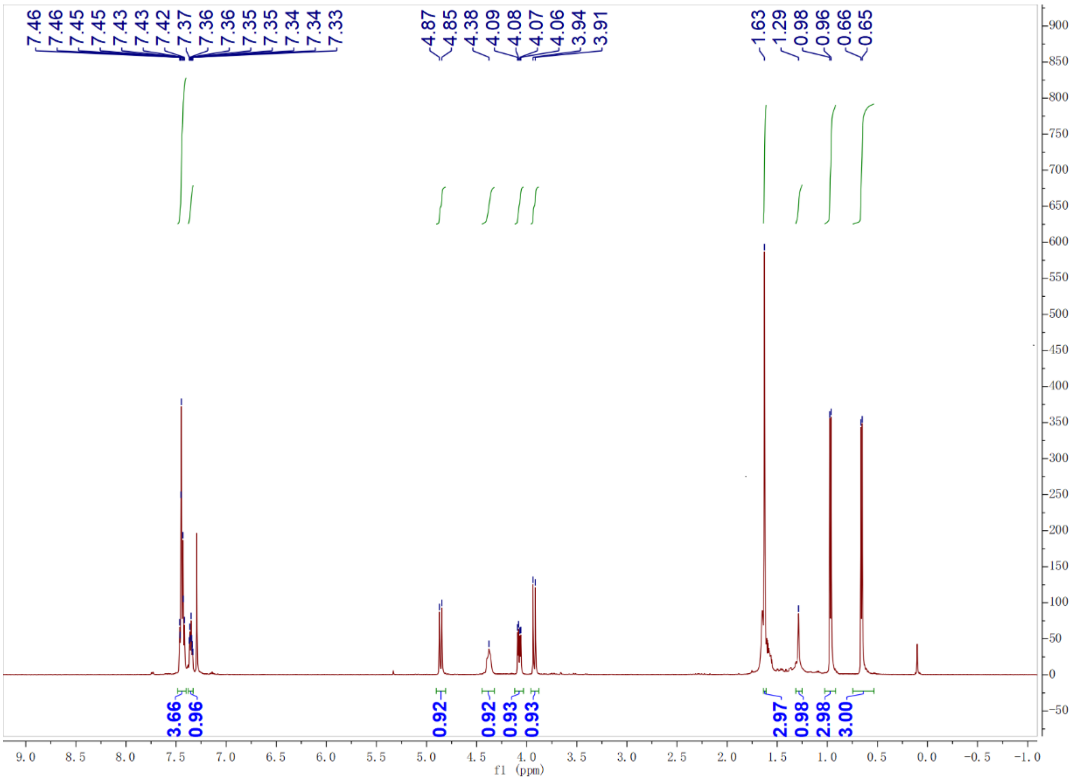

^{13}C NMR Spectrum of 7a

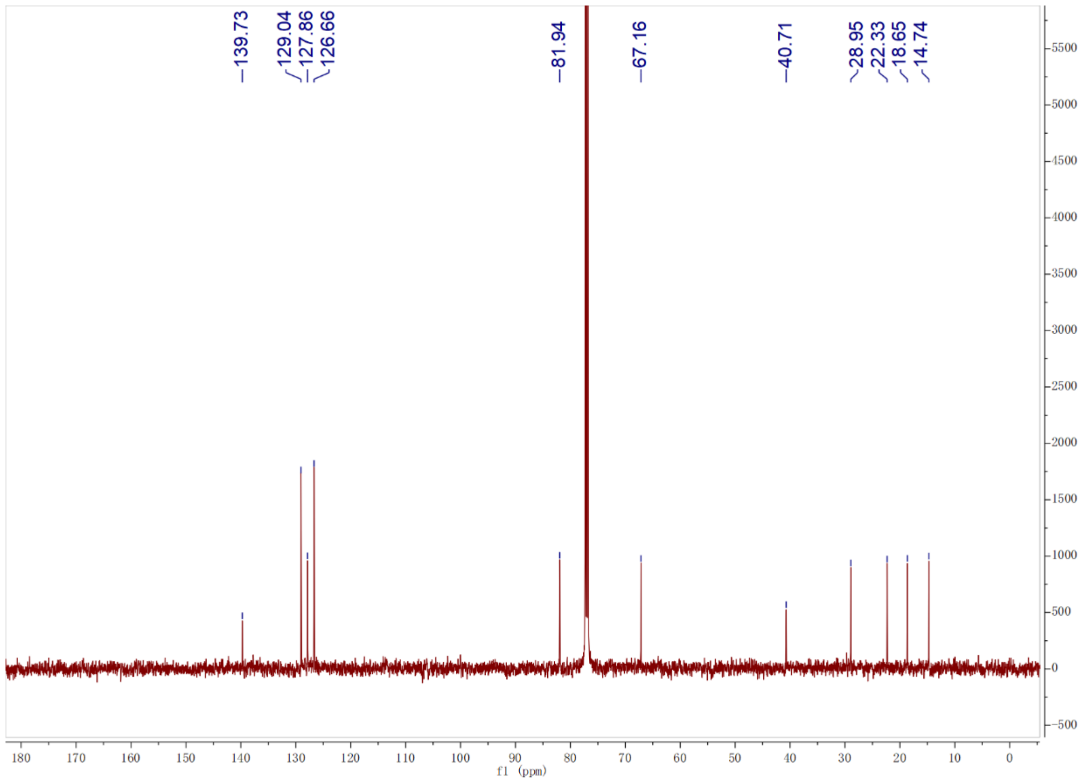

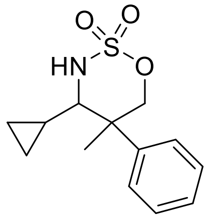

4-cyclopropyl-5-methyl-5-phenyl-1,2,3-oxathiazinane 2,2-dioxide (**8a**)

80% yield, dr>20:1, yellow oil. ^{1}H NMR (500 MHz, DMSO-d6) δ 7.84 (d, J = 9.7 Hz, 1H), 7.47 – 7.28 (m, 5H), 4.72 (d, J = 11.5 Hz, 1H), 4.10 (d, J = 11.4 Hz, 1H), 3.24 (t, J = 9.3 Hz, 1H), 1.59 (s, 3H), 0.71 (dt, J = 8.9, 4.7 Hz, 1H), 0.36 – 0.23 (m, 1H), 0.16 (dd, J = 9.8, 5.0 Hz, 1H), 0.06 – -0.06 (m, 1H), -0.62 (dd, J = 9.7, 4.9 Hz, 1H). ^{13}C NMR (126 MHz, DMSO-d6) δ 138.10, 126.24, 124.99, 124.74, 77.74, 64.84, 38.13, 12.26, 8.40, 1.66.; HRMS (ESI-TOF^{+}): m/z Calcd. for $C_{13}H_{17}NNaO_4S$ [(M+Na)$^{+}$]: 290.0827. Found: 290.0828

^{1}H NMR Spectrum of 8a

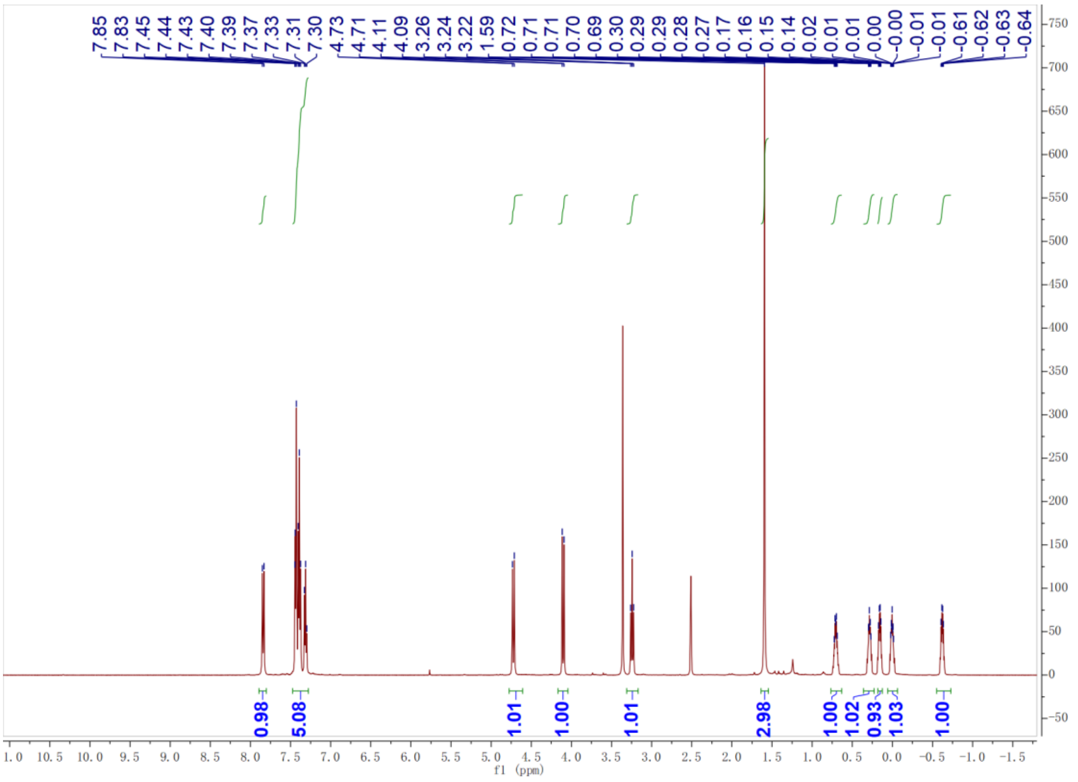

^{13}C NMR Spectrum of 8a

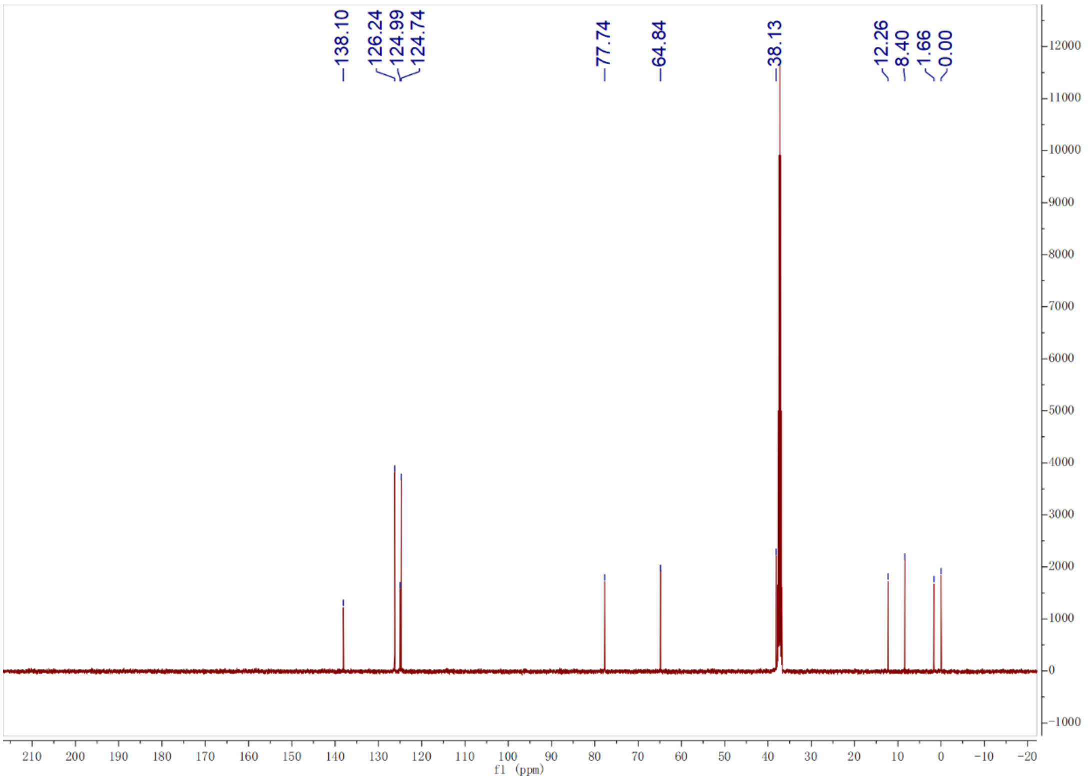

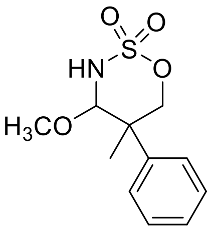

4-methoxy-5-methyl-5-phenyl-1,2,3-oxathiazinane 2,2-dioxide(**9a**)

81% yield, dr>20:1, yellow oil. ^{1}H NMR (500 MHz, DMSO-d6) δ 8.23 – 8.12 (m, 1H), 7.52 – 7.44 (m, 2H), 7.40 (dd, J = 8.7, 6.8 Hz, 2H), 7.35 – 7.27 (m, 1H), 4.99 (dd, J = 8.3, 2.0 Hz, 1H), 4.50 (d, J = 12.0 Hz, 1H), 4.37 (d, J = 11.8 Hz, 1H), 3.34 (s, 3H), 1.40 (s, 3H). ^{13}C NMR (126 MHz, DMSO-d6) δ 139.56, 127.99, 127.89, 126.62, 126.11, 91.00, 75.60, 55.35, 40.70, 14.90; HRMS (ESI-TOF^{+}): m/z Calcd. for $C_{11}H_{15}NNaO_4S$ [(M+H)$^{+}$]: 280.0613. Found: 280.0619

^{1}H NMR Spectrum of 9a

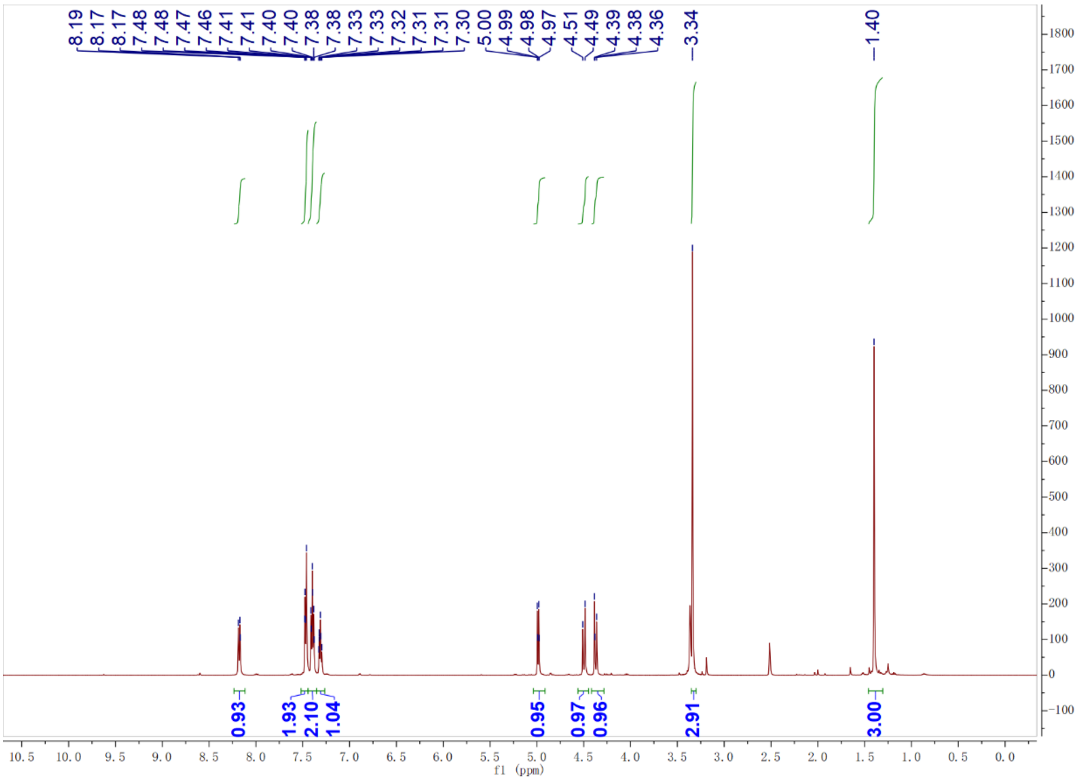

^{13}C NMR Spectrum of 9a

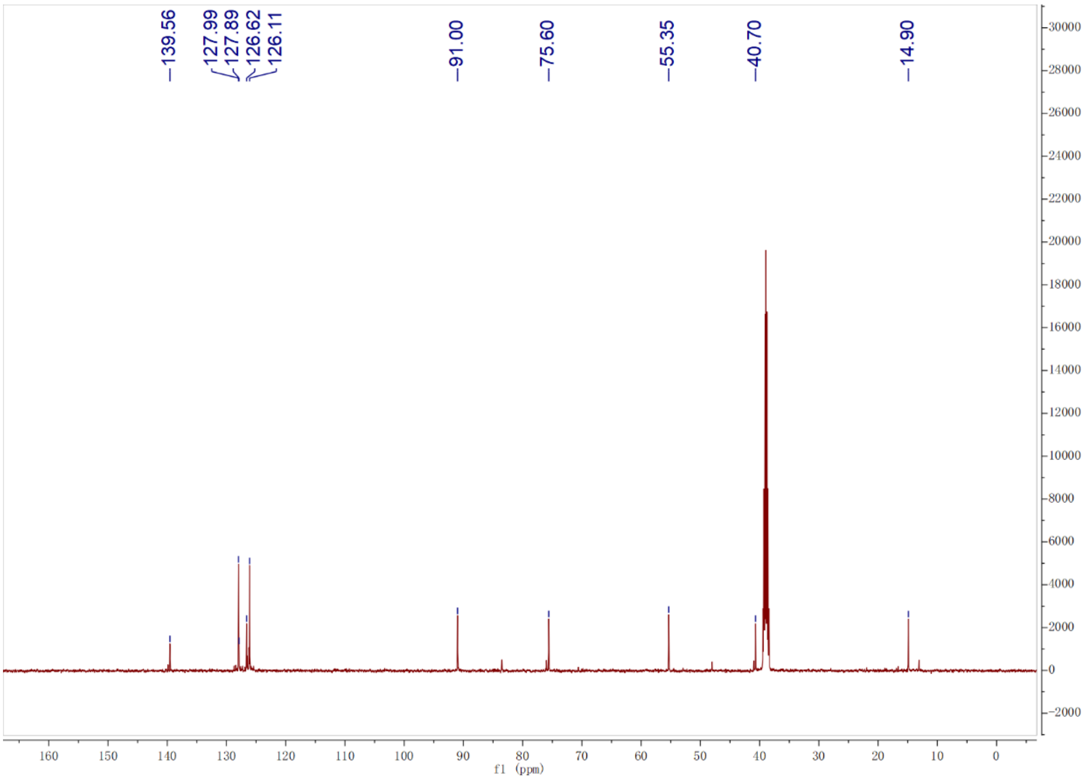

.

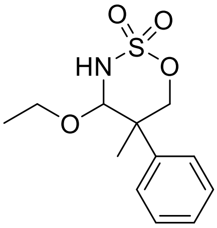

4-ethoxy-5-methyl-5-phenyl-1,2,3-oxathiazinane 2,2-dioxide（**10a**）

85% yield, dr>20:1, yellow oil. ^{1}H NMR (500 MHz, Chloroform-d) δ 7.32 (d, J = 6.1 Hz, 4H), 7.25 (dq, J = 6.0, 2.8 Hz, 1H), 4.97 (d, J = 9.3 Hz, 1H), 4.67 (d, J = 12.0 Hz, 1H), 4.38 (s, 1H), 4.12 (d, J = 11.9 Hz, 1H), 3.75 (dq, J = 10.1, 7.1 Hz, 1H), 3.37 (dq, J = 10.2, 7.1 Hz, 1H), 1.45 (s, 3H), 0.93 (t, J = 7.1 Hz, 3H). ^{13}C NMR (126 MHz, Chloroform-d) δ 137.96, 127.83, 126.78, 125.45, 90.11, 64.27, 40.57, 13.54, 13.04. HRMS (ESI-TOF^{+}): m/z Calcd. for $C_{12}H_{17}NNaO_4S$ [(M+Na)$^{+}$]: 294.0776. Found: 294.0754

^{1}H NMR Spectrum of 10a

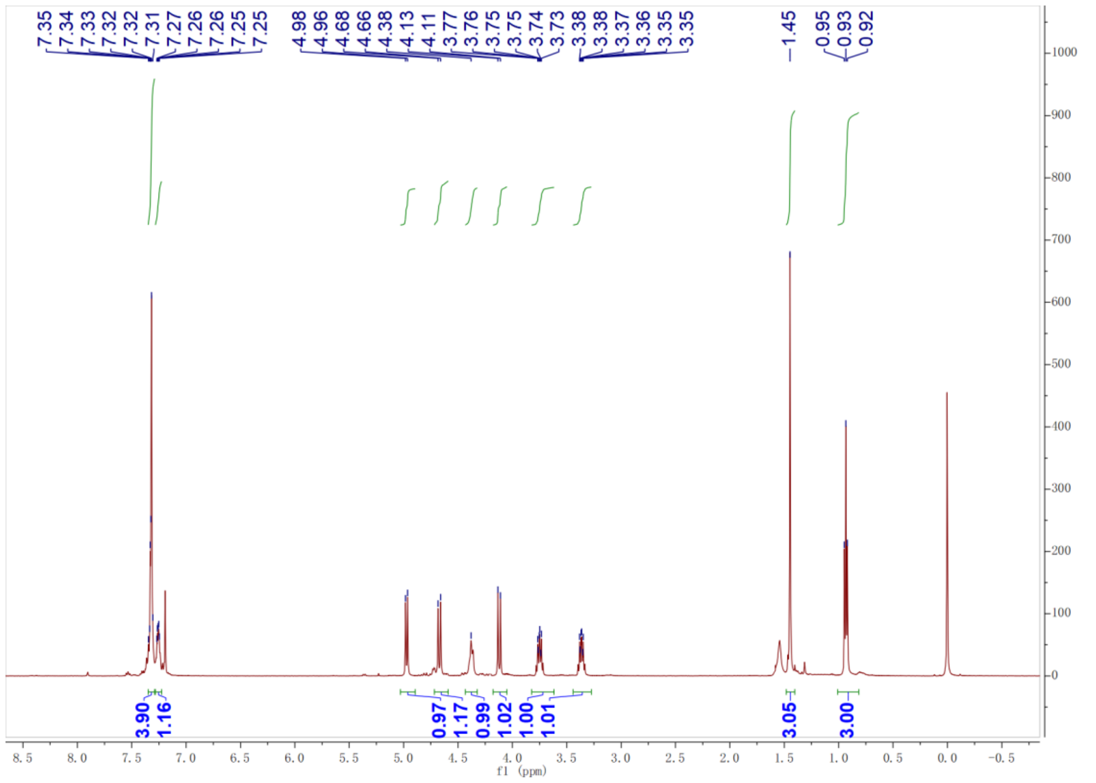

^{13}C NMR Spectrum of 10a

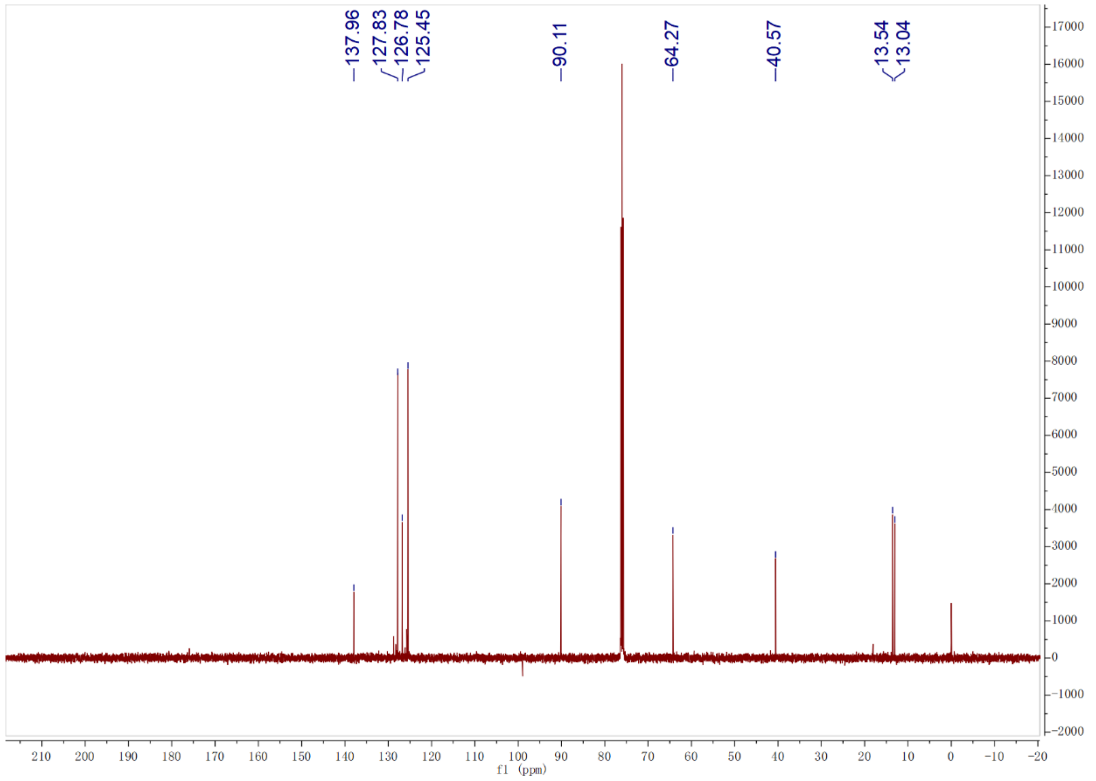

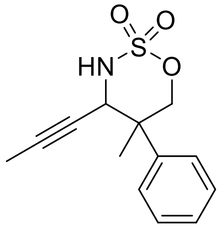

5-methyl-5-phenyl-4-(prop-1-yn-1-yl)-1,2,3-oxathiazinane 2,2-dioxide（**11a**）

78% yield, dr>20:1, yellow oil. ^{1}H NMR (500 MHz, DMSO-d6) δ 8.25 (d, J = 9.5 Hz, 1H), 7.44 – 7.34 (m, 2H), 7.31 (dd, J = 8.5, 6.9 Hz, 2H), 7.26 – 7.16 (m, 1H), 4.74 (dd, J = 9.6, 2.5 Hz, 1H), 4.50 (d, J = 11.7 Hz, 1H), 4.17 (d, J = 11.7 Hz, 1H), 1.59 (d, J = 2.4 Hz, 3H), 1.51 (s, 3H). ^{13}C NMR (126 MHz, DMSO-d6) δ 139.35, 128.61, 127.48, 126.76, 82.51, 78.88, 73.62, 55.13, 15.44, 3.00.; HRMS (ESI-TOF^{+}): m/z Calcd. for $C_{13}H_{15}NNaO_3S$ [(M+Na)$^{+}$]: 288.0670. Found: 288.0671.

^{1}H NMR Spectrum of 11a

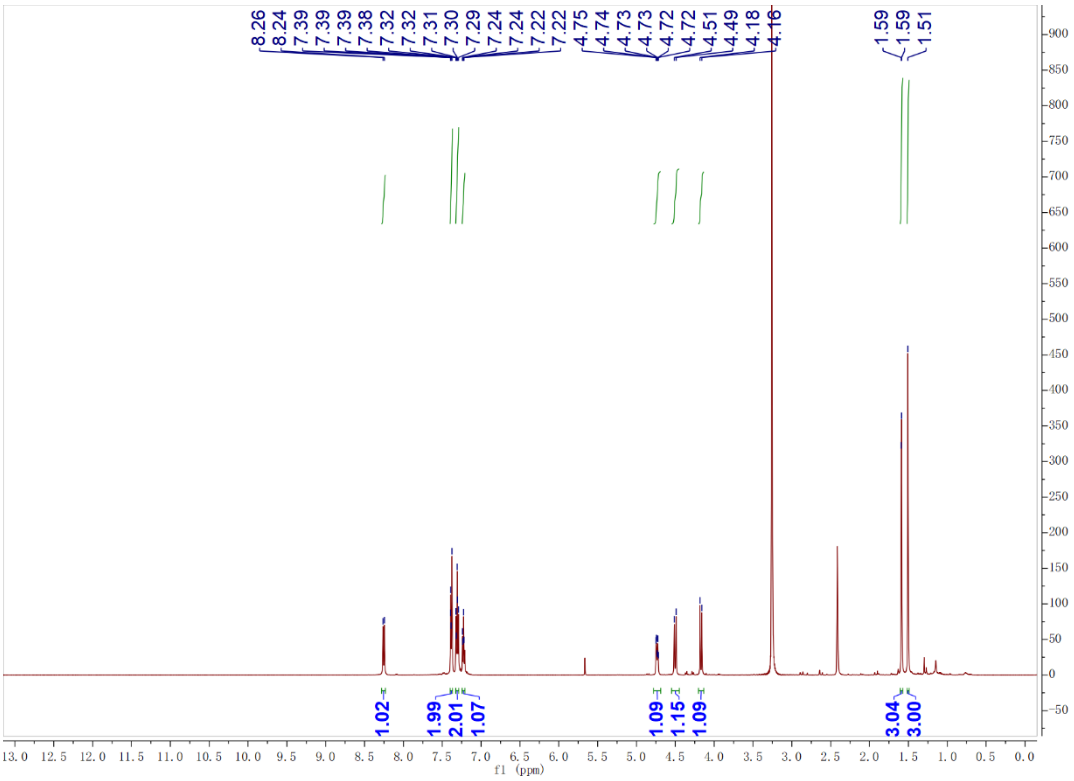

^{13}C NMR Spectrum of 11a

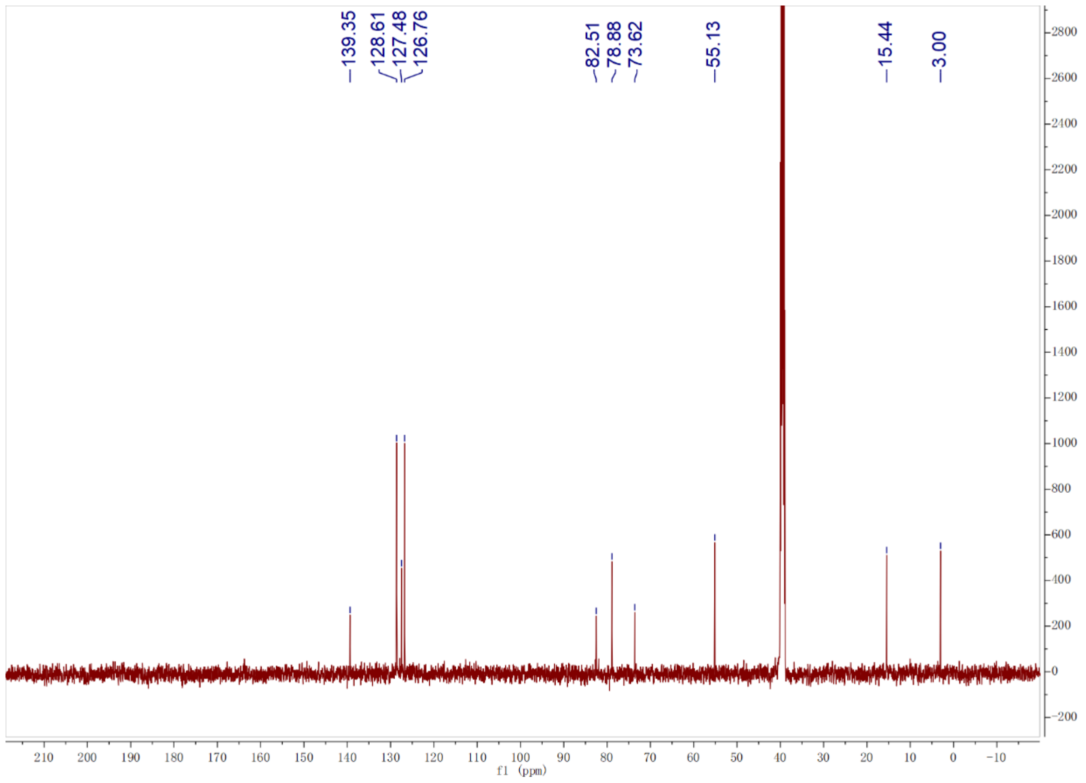

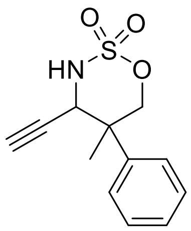

4-ethynyl-5-methyl-5-phenyl-1,2,3-oxathiazinane 2,2-dioxide（**12a**）

75% yield, dr>20:1, yellow oil. ^{1}H NMR (500 MHz, DMSO-d6) δ 8.50 (d, J = 9.7 Hz, 1H), 7.53 – 7.47 (m, 2H), 7.41 (dd, J = 8.6, 6.9 Hz, 2H), 7.34 (dd, J = 7.7, 1.6 Hz, 1H), 4.92 (dd, J = 9.6, 2.5 Hz, 1H), 4.63 (d, J = 11.6 Hz, 1H), 4.30 (d, J = 11.7 Hz, 1H), 3.38 (d, J = 2.4 Hz, 1H), 1.63 (s, 3H). ^{13}C NMR (126 MHz, DMSO-d6) δ 138.40, 128.11, 127.06, 126.31, 78.42, 77.42, 76.89, 54.23, 14.79.; HRMS (ESI-TOF^{+}): m/z Calcd. for $C_{12}H_{13}NNaO_{3}S$ [(M+Na)$^{+}$]: 274.0514. Found: 274.0533.

^{1}H NMR Spectrum of 12a

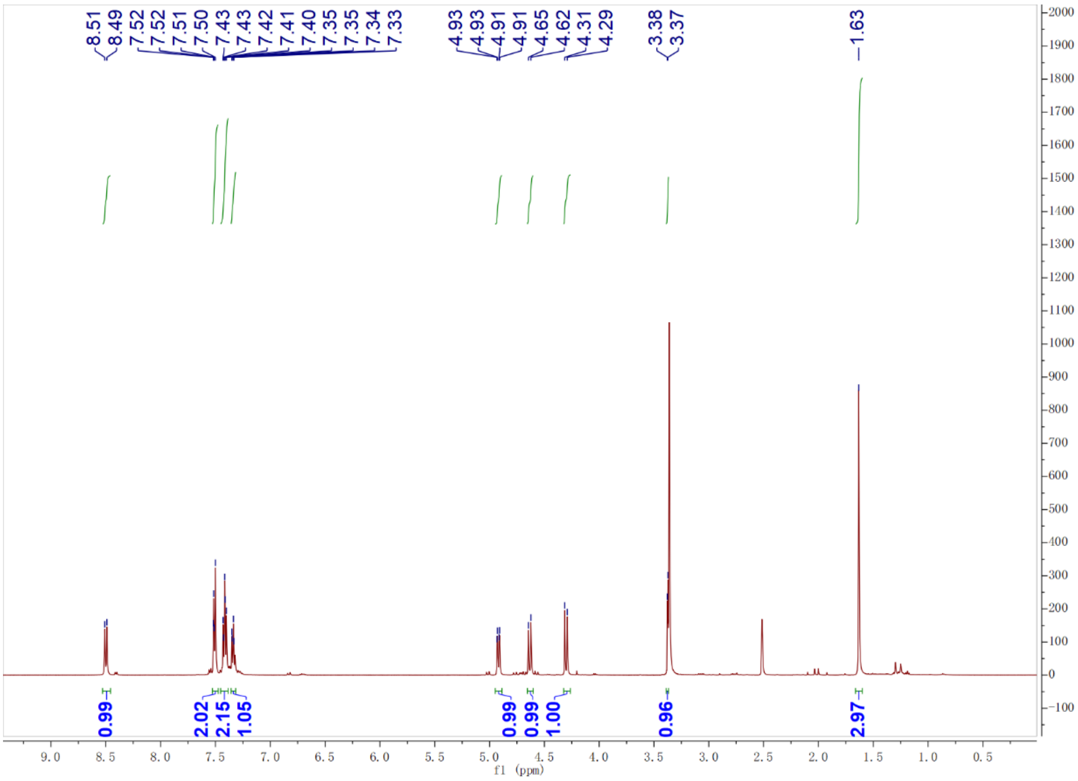

^{13}C NMR Spectrum of 12a

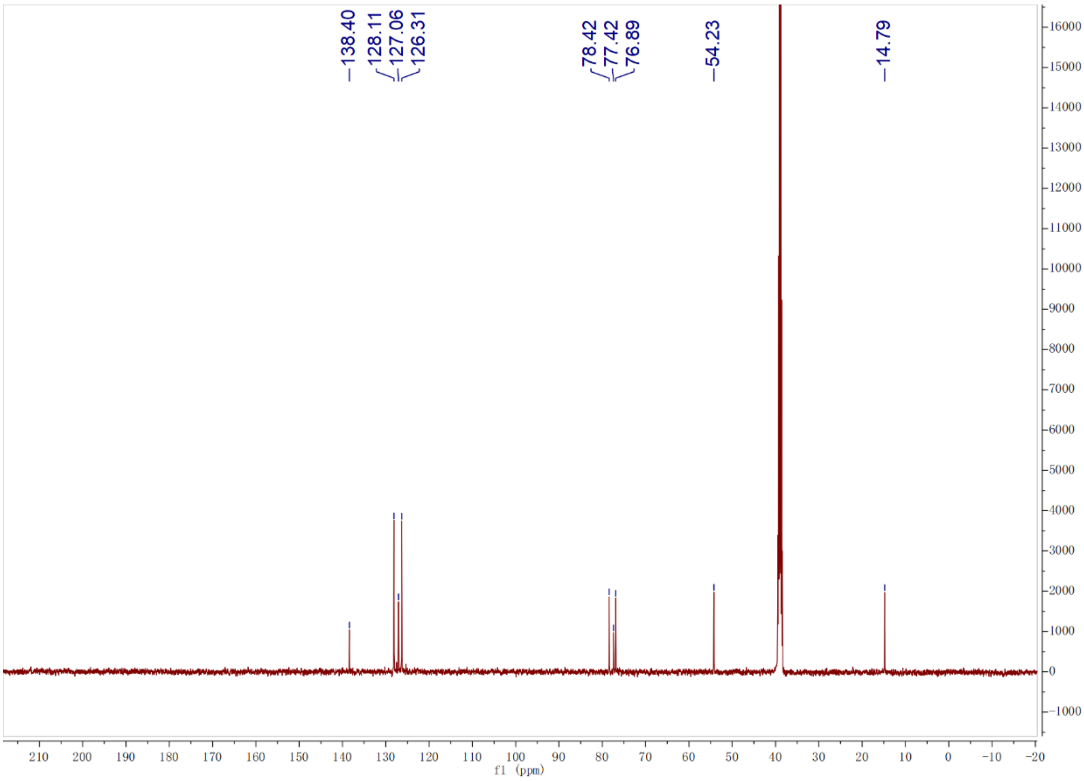

Supplement: Supplementary file 1 [file molecules-27-06174-s001.zip › molecules-1903085-supplementary.pdf]
